# Electrochemical organic reactions: A tutorial review

**DOI:** 10.3389/fchem.2022.956502

**Published:** 2023-01-10

**Authors:** Joyeeta Lodh, Shounik Paul, He Sun, Luyang Song, Wolfgang Schöfberger, Soumyajit Roy

**Affiliations:** ^1^ Eco-Friendly Applied Materials Laboratory (EFAML), Materials Science Centre, Department of Chemical Sciences, Mohanpur Campus, Indian Institute of Science, Education and Research, Kolkata, West Bengal, India; ^2^ Institute of Organic Chemistry, Laboratory for Sustainable Chemistry and Catalysis (LSusCat), Johannes Kepler University (JKU), Linz, Austria

**Keywords:** electrocatalysis, organic reaction, CO_2_ reduction, kinetics, electrochemical techniques and methods

## Abstract

Although the core of electrochemistry involves simple oxidation and reduction reactions, it can be complicated in real electrochemical organic reactions. The principles used in electrochemical reactions have been derived using physical organic chemistry, which drives other organic/inorganic reactions. This review mainly comprises two themes: the first discusses the factors that help optimize an electrochemical reaction, including electrodes, supporting electrolytes, and electrochemical cell design, and the second outlines studies conducted in the field over a period of 10 years. Electrochemical reactions can be used as a versatile tool for synthetically important reactions by modifying the constant electrolysis current.

## 1 Introduction

Over the years, synthetic chemists have always faced the challenge of generating molecules with structures ranging from a few nanometers to increasingly bulky and complex structures in a sustainable way (Bryan et al., 2013; Kuttruff et al., 2014; Cernak et al., 2016). Owing to these challenges, the techniques and strategies are being constantly monitored to increase the chemical toolbox. These factors made photochemistry (Lodh et al., 2018; Mallick and Roy, 2018) and electrochemistry (Moeller, 2000; Sperry and Wright, 2006; Yoshida et al., 2008; Ogibin et al., 2009; Sequeira and Santos, 2009) re-emerge as an important route to synthesize industrially important precursor. The earliest mention of electrochemical methods could be dated back to the 19th century when the Hall–Heroult (Grjotheim et al., 1977) and Chloralkali processes (Lakshmanan and Murugesan, 2014) were developed to perform the electrolysis of aqueous sodium chloride and Al_2_O_3_, respectively. However, despite the huge popularity of electrochemical reactions to synthesize valuable chemicals on a metric-ton scale, the technique has rarely been used in organic chemistry for the synthesis of value-added chemicals. The electrochemical pathway is advantageous in many ways over the traditional pathway owing to its mild condition, tolerance for a functional group, sustainability, and easy scalability. The major challenges to adapting the electroorganic reactions include 1) separation of product, which is an issue for both conventional and electroorganic synthesis; 2) reaction setup, which is critical to the electroorganic reaction; and 3) the choice of the solvent, which influences the ionic conductivity in the reaction setup. The major challenges to adapting the electroorganic reactions include 1) separation of product, which is an issue for both conventional and electroorganic synthesis; 2) reaction setup, which is critical to the electroorganic reaction; and 3) the choice of the solvent, which influences the ionic conductivity in the reaction setup. It is important to realize that any chemical reaction will have its electrochemical counterpart. The reverse is also true, provided a distinct catalyst is known. However, one must realize that these two techniques are presently non-parallel and instead appear to be complementary. This could be changed with increased knowledge of factors influencing the chemical and electrochemical pathways. The advantages could be numerous once there are multiple parameters for the electrochemical pathway. The advantages could be 1) yield (in major cases adequate or ample); 2) negligible cost (excluding the cost of equipment, the chemical reagent and power are relatively cheap); and 3) easy work-up (the formation of side product is little, and in most cases, work-up involves the removal of only the electrolyte and solvent). Numerous review articles have been published in this field of organic electrochemistry that summarize the advances made over the years, such as the Kolbe reaction (Glasstone and Hickling, 1939; Vijh and Conway, 1967) and electrochemical reduction (Popp and Schultz, 1962; Elving and Pullman, 2009). The groups of Steckhan (Steckhan, 1986; Steckhan, 1987), Little (Francke and Little, 2014), and Nikishin (Ogibin et al., 2009) reviewed the advances in indirect electrolysis. The cathodic reduction and anodic oxidation processes were reviewed in detail by the groups of Yoshida (Yoshida et al., 2008), Schäfer (Schäfer, 1981; Schäfer, 2011), Wright (Sperry and Wright, 2006), and Tian (Chen et al., 2019). The anodic electrochemical process was also reviewed by the groups of Eberson (Eberson and Nyberg, 1973; Eberson and Nyberg, 1976), Shono (Shono, 1984), Adams (Adams, 1969), Schäfer (Schafer et al., 2007; Schafer, 2014), Chiba (Chiba and Kim, 2009), Moeller (Moeller, 1997; Moeller, 2000; Moeller, 2016; Feng et al., 2017), and Boydston (Ogawa and Boydston, 2015). The group of Waldvogel (Möhle et al., 2018; Waldvogel et al., 2018; Wiebe et al., 2018; Schulz and Waldvogel, 2019), Bao Guo Sun (Yang Q. L. et al., 2018), Tian-Sheng Mei (Ma et al., 2018), Zeng (Jiang et al., 2017), Zhang (Guo et al., 2015), and Daugulis (Daugulis et al., 2015) summarized the synthetic attempts at complex structures using electrochemistry. There were also detailed reviews on electrochemical alkyne functionalization by the Nisar Ahmed group (Martins et al., 2019). This review is divided into two major themes. In the first part, we focus on learning the basics of electrochemistry such that at any given point, for an electrochemical system, the relation between the current and voltage could be exploited to understand the behavior of an electrochemical cell and improve its catalytic performance. The second part focuses on synthetic electrochemical organic transformations on a regular laboratory scale with a special focus on the choice of electrodes. The examples have been chosen in a manner to provide exhaustive coverage of the electroorganic reactions (Table 1), at the same time eliminating an ambiguous example pertaining to selectivity, yield, and other parameters.

**TABLE 1:** Comparing different reaction strategies for electroorganic synthesis.

Category	Working electrode	Counter electrode	Reaction	Potential	References
C-N functionalization	Glassy carbon disk electrode	Platinum wire	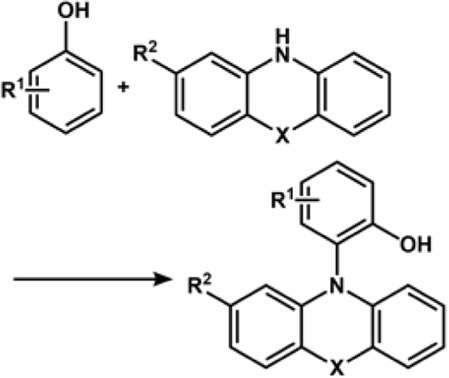	0.70 V	Tang et al. (2018b)
C-C coupling	Glassy carbon electrode tip	Glassy carbon rod	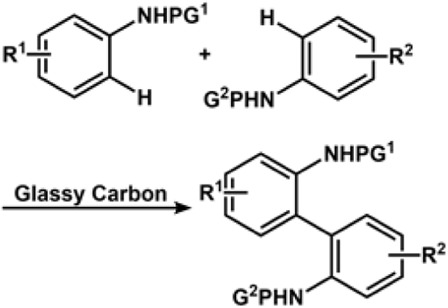	1.39 V	Waldvogel et al. (2018)
C-H oxygenation	Glassy carbon/RVC/carbon paper	Platinum foil	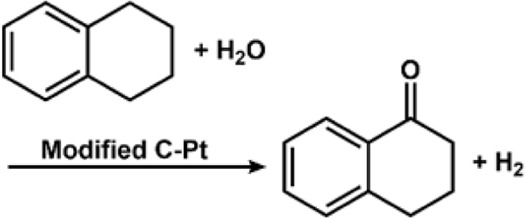	1.6 W	Ding et al. (2016)
Annulation	Glassy carbon	Platinum wire	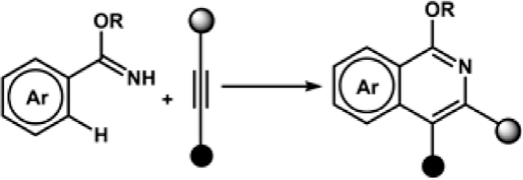	1.5 V	Kong et al. (2019)
Halogenation	Glassy carbon	Platinum wire	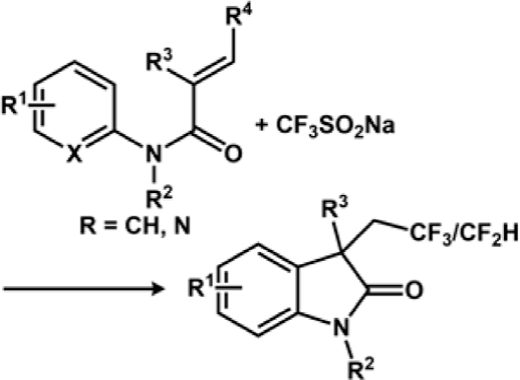	1.1 V	Ruan et al. (2019)
Electrochemical CO_2_ reduction	Cobalt (III)-corrole immobilized carbon fiber paper	Pt wire	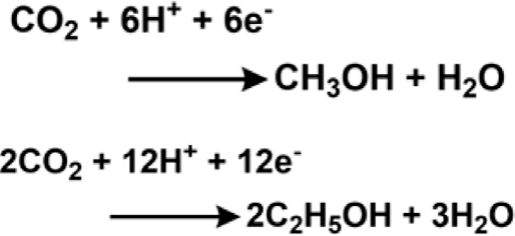	−0.80v	Gonglach et al. (2019)
Electrochemical CO_2_ reduction	Manganese (III)-corrole immobilized carbon fiber paper	Pt wire	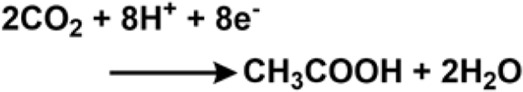	−1.25 V	De et al. (2020)

In this review, we not only attempt to present an overview of multiple parameters that govern the electrochemical pathway. We also focus a great deal on the types of organic reactions conducted over a period of 5 years. These reactions will be segregated according to the electrochemical methods employed. This review aims to help the researchers understand and adopt electrochemical pathways in a general synthetic organic reaction.

## 2 Basic requirements for electroorganic synthesis

In this section, we delve into the basics of electrochemistry, which might help a novice to foray into the field of electrochemistry. It is important to note the parameters which help distinguish any electrochemical organic reaction from a general organic reaction. The driving force for any redox reaction is potential, often referred to as voltage. One of the most important parameters would be a typical power source connected to three electrodes, namely, cathode, anode, and reference. When attached to an electrochemical cell, the power source controls the potential between the reference and working electrodes. The reaction of interest occurs at the working electrode, whereas the other could be referred to as the counter electrode. The anode and cathode can be designated as working electrodes. The potential difference between the cathode and anode would be driven by the power source in case a reference electrode is used. In order to create an oxidative environment at the anode and a reductive environment at the cathode, the power source would push electrons in the cathode from the anode. Another important aspect of any electrochemically driven reaction would be current, which is indicative of the rate of electron flow, is impossible to disentangle from voltage, and facilitates redox reaction. The fourth and final parameter would be the reaction medium containing an electrocatalyst, which acts as a circuit through which charged species move.

Before discussing the reaction types in an electrochemical cell, it is important to discuss what constitutes an electrochemical cell. The equipment for electrochemical cells could range from a simple beaker to elaborate instruments. However, in the interest of electroorganic synthesis, capacitors, redox-flow cells, and batteries would not be discussed.

### 2.1 Power supply

A simple method to ensure a supply of electricity would be attaching a suitable battery with a suitable potential, which helps overcome the resistance of an electrochemical cell. The power source could come in various forms. In electroorganic conversion, one might often encounter experiments conducted by potentiostats or galvanostats.

### 2.2 Electrodes

One of the most important constituents of an electrochemical organic synthesis is the choice of the electrode (Hilt, 2020) with regard to the surface area, reusability, and constituents. The major challenges in the field of electrochemistry would be the availability of the non-standardized nature of electrodes, including the myriad of semiconducting materials from the supermarket, such as aluminum foil. The choice of electrode material would significantly impact the fate of the reaction because the transfer of electrons occurs at the surface of the electrode. It is required to incorporate a “reference electrode.” However, the reference electrode is not a necessity for preparative constant potential experiments. It is important to gauge the adsorption of active species on the electrode surface and the diffusion of reactive species into the solution. The mode of operation could always be referred to from the literature.

### 2.3 Solvents and electrolytes

In an electrochemical cell, the reducing resistance or increasing conductivity is often dictated by solvents and electrolytes. Organic solvents employed in electroorganic conversions are often polar aprotic, which dissolves the substrates, electrolytes, and reactants. There have been numerous reviews on solvents and their properties for electrochemical reactions. This section briefly discusses solvents and their properties that make them relevant for electrochemical reactions (Mann and Grunwald, 1959; Sawyer et al., 1995; Lund and Hammerich, 2001; Janz, 2012; Kissinger and Heineman, 2018).

One of the important properties of an electrochemical solvent is its ability to dissolve and dissociate ionic salts to achieve high ionic conductivity. However, an exception to this rule is electrochemically induced precipitation or electrodeposition, wherein the solvation of solvents and products by the solvent is desired. Other properties of the electrochemical solvent include its polarity, oxidation/reduction property, acidity/basicity, and electrophilic/nucleophilic behavior. The susceptibility of solvent toward oxidation/reduction is a critical point because a solvent is often exposed to electrodes at high oxidizing or reducing potentials (Bockris et al., 1992; Conway and Bockris, 2012). In order to maintain the charge neutrality of the cell, the presence of charged species (electrolytes) is important. Electrolytes also help in the modification of the surface of the electrode during the reaction. The electrolytes can range from ionic liquids to polar solvents, which are conducive due to the presence of soluble organic salts, such as Bu_4_NF_4_.

### 2.4 Electrochemical cell design

The designing of an electrochemical cell depends entirely on the nature of the experiments. The materials for designing the electrochemical cell could vary from glass (e.g., quartz or Pyrex) to nylon or Teflon. (White, 2012). However, an electrochemical cell should be designed keeping in mind the following points: 1) it should be cost-effective and 2) it must be inert toward the electrochemical reaction. Additionally, it is important to note that the material used for making the electrochemical cell must not interfere during sensitive measurements. For example, systems involving hydrofluoride or high pH can lead to the corrosion of glass. Similarly, in the presence of an organic solvent, a cell made of plastic might decompose. This section provides a brief description of the two-electrode cell, three-electrode cell, and electrochemical cell used for specialized purposes.

#### 2.4.1 Undivided cell

**FIGURE 1:**
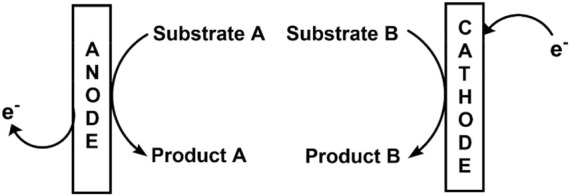
Schematic for an undivided electrochemical cell.

**FIGURE 2:**
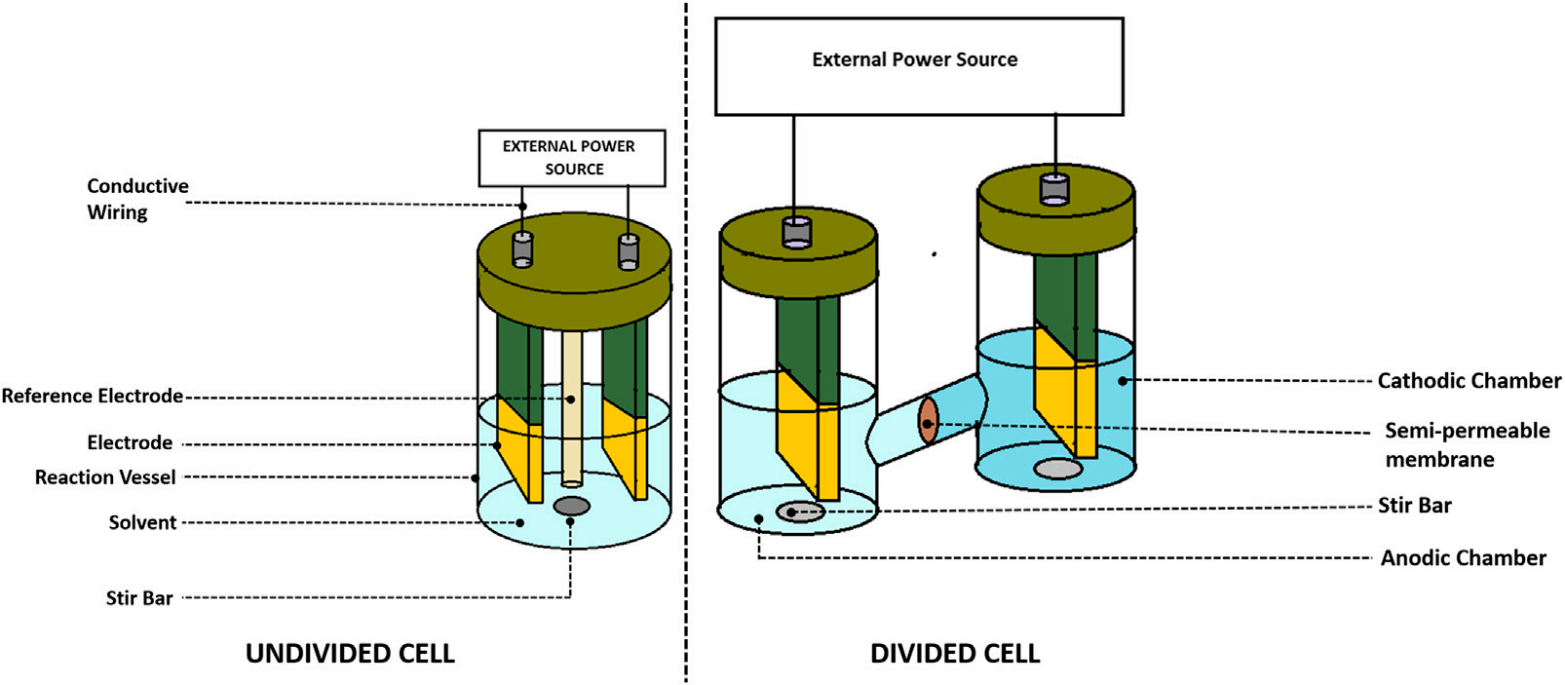
The undivided and divided cell configuration of electrochemical cell used in electroorganic synthesis.

**FIGURE 3:**
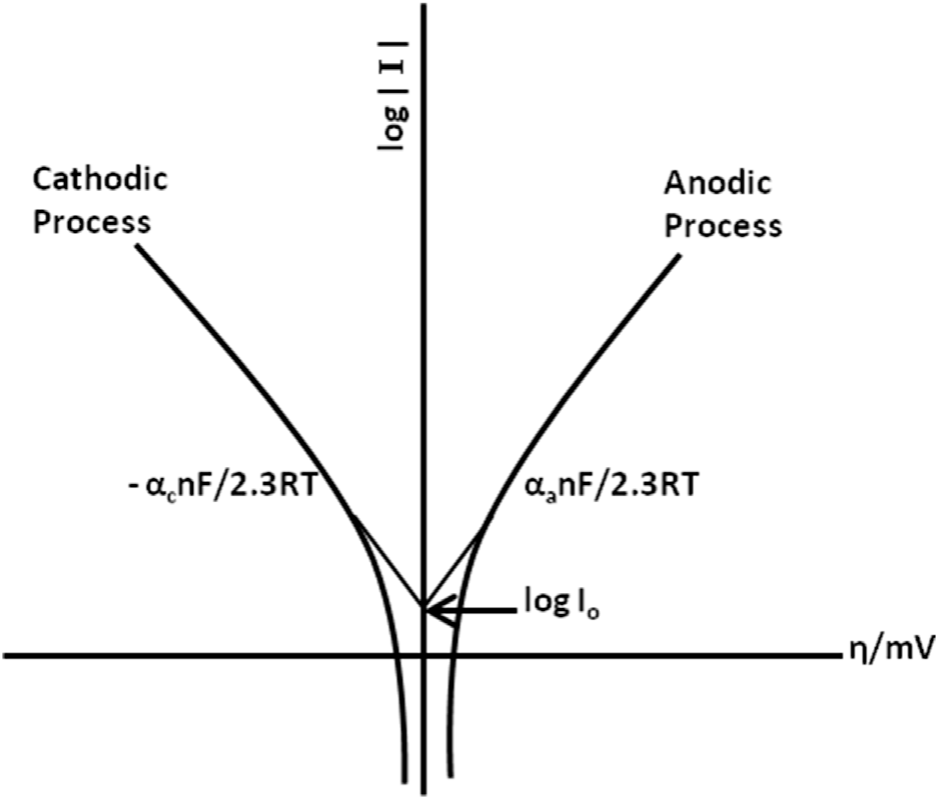
Schematic for a divided electrochemical cell.

In order to assemble an undivided cell (Ma et al., 2021) as shown in Figures 1, 2, it is important to ensure that 1) the electrodes do not touch each other and 2) there is a distance between the electrode holders and electrodes. To create an inert atmosphere, the beaker-type cell could be sealed with a Teflon stopper. The bulk electrolysis at the undivided cell not only transforms substrate A but also could make a change in the material at the counter electrode. It is important to ensure that product A should not convert back to substrate A at the counter electrode causing the electrolysis to become inefficient (Figure 3).

#### 2.4.2 Divided cell

The divided cells (Pollok and Waldvogel, 2020) are more elaborate than the undivided cells (see in Figures 2, 3). They are separated using a porous membrane, which allows conductivity. There is a necessity for a separator in a two-compartment cell to concur the further reaction of substrate A at the counter electrode due to byproduct electrolysis such as product B. It is also feasible that the productive electrolysis occurs at the counter electrode. The electric current could be used at the electrode (paired electrolysis) because there is no issue of separation between product A and product B.

##### 2.4.2.1 Two-electrode electrochemical cell

The two-electrode electrochemical cell (Grimshaw, 2000; Frontana-Uribe et al., 2010) consists of a working electrode and a reference electrode wherein the potential of the reference electrode is monitored at a constant value and the potential of the working electrode is measured with respect to the reference electrode. The reference electrode can be considered an ideal non-polarized electrode as the current passes through the working and reference electrodes. The two-electrode electrochemical cells have been used in polarographic studies. In such studies, the dropping mercury electrode (DME) usually acts as the working electrode and a large mercury pool acts as the reference electrode. The area of the pool is substantially higher than that of the working electrode (DME). Thus, the pool can be regarded as unpolarized, making it a good reference for potential control. Solution resistance, R_s_, and the ***i*** R_s_ drop are important parameters that should be monitored. Non-aqueous mediums are usually more resistive as a system. In order to cope with it, ultramicroelectrodes can be used because they have a quite low current profile in the range of nA. This low current profile helps with solutions providing resistance in the scale of kΩ to MΩ.

##### 2.4.2.2 Three-electrode electrochemical cell

The three-electrode electrochemical cells (Kingston et al., 2019) are majorly used in studies wherein the cell resistance is high. Although the potential of the working electrode is measured with respect to the reference electrode, the current over here passes through the working electrode and a counter electrode (auxiliary). The reference electrode, in this case, approaches ideal non-polarizability because no current passes through it. Hence, it is more reliable for monitoring the potential. During the experiments, the tip of the reference electrode is kept in the vicinity of the working electrode to minimize solution resistance. It is important to ensure that the tip of the reference electrode must not interfere with the mass transfer of electrolyte species. In most electrochemical experiments, three electrodes can be placed together in one compartment. However, under special conditions, it might be necessary to isolate the reference or auxiliary electrode from the solution containing the working electrode. We can choose a reference electrode based on the electrochemical reactions we perform (Inzelt et al., 2013). The incompatibility of the reference electrode with that of the system can lead to clogging of the reference electrode, precipitation of the charged species with poor solubility in the frit, and an increase in junction potential. Temperature and pressure are also important parameters while deciding on the reference electrode. The time of the experiment is also a limiting factor while deciding on the reference electrode because using a less robust reference electrode, in this case, can result in the stability of the reference electrode over a period of time. For the reference electrode to be flexible, a double junction or a salt bridge could be incorporated, which separates the reference electrode from the working electrode in case of fixed potential.

#### 2.4.3 Quasi-divided cell

The quasi-divided cell merges the feature of the undivided and divided electrochemical cells. Usually, the quasi-divided cell (Senboku et al., 2022) constitutes electrodes with two different surface areas and galvanostatic electrolysis under a low current. The two different electrodes are subjected to different environments.

##### 2.4.3.1 Small surface counter electrode

Owing to the small surface area of the electrode, the current density is high at the small surface counter wire electrode and flows in the opposite direction. The concentration for the starting material is low, as is the mass transfer to the electrode. Hence, the solvent gets electrolyzed due to a large amount of current.

##### 2.4.3.2 Large surface working electrode

The low constant current at the working electrode causes low current density. As a result, the substrate with the lowest redox potential would get electrolyzed during the entire course of quasi-potentiostatic electrolysis. Usually, the undesired side reaction occurs only at the end.

#### 2.4.4 Separator (for the divided cell)

The cell compartment for the divided cell must be separated using a conductive material. The porous membrane should allow the transport of ions. However, the transport of reactants and products must be restricted. The separator could be chosen from materials such as polymer membranes to ceramic frits.

### 2.5 Types of reactions in an electrochemical cell

Considering two completely stable and soluble species, A and B, in an electrolysis medium consisting of an excess of electrolyte (which is electroinactive), the simplest electrode interconversion that could occur at the inert surface is

$$A+{ne}^{-}⇌B.$$

For the above electrode reaction to occur, it is important to ensure the supply of reactants to the surface of the electrode. Additionally, it is essential to remove the product for the electron transfer reaction to occur. For example, the reduction of A to B would involve the following steps:

$$A_{bulk}\overset{mass\;transport}{\to }\;A_{electrode},$$ (1)

$$A_{electrode}\overset{electron\;transfer}{\to }\;B_{electrode},$$ (2)

$$B_{electrode\;}\overset{mass\;transport}{\to }\;B_{bulk}.$$ (3)

The cathodic current and the rate of reduction can be gauged by the rate of the above-mentioned overall sequence, which also must be dependent on the slowest step. However, the electrode reactions are rarely this simple. They involve electron transfer at multiple steps (Marcus, 1959; Marcus, 1964; Hammes-Schiffer and Soudackov, 2008). It could broadly be categorized into three types based on electrode processes.

#### 2.5.1 Coupled chemical reactions

The chemical reactions, under favorable circumstances, can proceed *via* a single pathway generation of only one product. However, the organic reactions majorly follow competitive pathways leading to the formation of multiple products (Evans, 2008). For example, reducing p-iodonitrobenzene could lead to the generation of different products *via* different pathways.

##### 2.5.1.1 Chemical reaction could follow two pathways

1) Homogenous: the reaction would occur as a result of electron transfer and species B is transported away from the surface. 2) Heterogenous: species R is adsorbed on the surface.

It is rarely possible that the electroactive species formed during a chemical reaction is not the major species in bulk solution or that the reaction at the electrodes disturbs the equilibrium in the homogenous solution. For example, acetic acid would dissociate before getting reduced *via* the electron transfer:

$$CH_{3}COOH\;\to \;CH_{3}COO^{-}+H^{+},$$ (4)

$$H^{+}+e^{-}\to \;\frac{1}{2}H_{2}.$$ (5)

If the transport of acetic acid to the surface of the cathode is faster than the dissociation reaction, the current density will be limited due to the evolution of hydrogen.

#### 2.5.2 Adsorption

Adsorption plays an important role in the field of electrochemistry. During electrocatalysis, it is important for the intermediates to get adsorbed on the surface because it leads to a lower energy pathway (Eqs 1–3). In the adsorption pathway, electron transfer occurs at the surface of the electrode. However, there might or might not be any interaction between the surface of the electrode and the reagent (A or B). It is feasible to slow down the electron transfer reaction, change the product, or modify the electrode reaction by adsorption of species that might not be directly involved in the electron transfer process.

#### 2.5.3 Phase formation

There could also be a formation of a new phase through these electroreactions (Eq. 6), or one phase could get transformed into another. Eq. 6 includes steps such as diffusion, phase transformation, electron transfer, adsorption of intermediates, protonation, and hydration equilibria:

$$PbO_{2}+4H^{+}+SO_{4}^{2-}+2e^{-}\to PbSO_{4}+2H_{2}O.$$ (6)

However, a phase formation would require a multi-step process involving nucleation and subsequent growth. It is important to consider that the growth of a new phase could lead to the incorporation of metal ion atoms at an appropriate position in the lattice or diffusion of metal atoms due to the reduction of metal ions in the solution.

### 2.6 Coulombic efficiency

For an electrochemical reaction, an ionically conductive reaction medium is required. The energy efficiency of an electroorganic synthesis could be determined using the electrolytic conductivity, ĸ , of the reaction medium and the selectivity and energy efficiency of the reaction medium (Harnisch and Schröder, 2019). Coulombic efficiency, for an electrochemical system, can be defined as the ease with which charge can be transferred to drive an electrochemical reaction. It is quantified using the following formula:

$$nC=\frac{Q_{discharge}}{Q_{charge}}\times 100,$$

where Q is the amount of charge passed.

With the increase in the number of cycles, *nC* decreases.

### 2.7 Classical electrochemical techniques

This section discusses the classical electrochemical experiments to help the readers choose the techniques suitable for their experiments. The classical techniques have been chosen for their availability in commercial instruments. Electrochemical processes involve diffusion-controlled mass transport, facilitated by 1) applying controlled hydrodynamics that acts as a limiting factor to diffusion layer thickness; 2) using a large concentration of electrolyte to limit the migration of mobility of ions in the electric field (within the working and counter electrodes); 3) limiting the interference of natural convention by completing the experiment in the shortest time possible; 4) introducing a steady state diffusion controlled current by using microelectrodes before the interference of microelectrodes.

Electrochemical techniques are considered versatile, precise, and powerful owing to their large linear dynamic range and comparatively low-cost instruments. Most of the electrochemical techniques are based on the concept of measuring the resultant current due to the continuously changing applied potential at the electrode solution interface. Modern electrochemical techniques include stripping analysis which involves concentrating the analyte into or onto the surface of the electrode. There are different forms of stripping analysis (Kalvoda and Kopanica, 1989; Farghaly et al., 2014), such as anodic stripping voltammetry (ASV), cathodic stripping analysis (CSV), adsorptive stripping voltammetry (AdSV), and potentiometric stripping analysis (PSA). The modern variation of stripping analysis appeared first with fast linear sweep voltammetry, followed by the development of square wave and pulse polarography.

Another rapid voltage scan technique is cyclic voltammetry (Joseph, 2000), in which the direction of the voltage scan is reversed. The resulting current is recorded at the applied potential of the working electrode in forward and reverse directions. The parameters to consider in cyclic voltammetry are 1) anodic and cathodic peak current (I_pa_ and I_pc_); 2), anodic and cathodic peak potential (E_pa_ and E_pc_); and 3) half peak potential where cathodic and anodic currents reach their half value (E_p/2_).

Pulsed voltammetry is a series of potential pulses with increasing amplitudes. The peak current is recorded at the end of each pulse. Different forms of pulsed voltammetry (Barker and Gardner, 1960) include normal pulse voltammetry (NPV) and differential pulse voltammetry (DPV).

Square wave voltammetry (SWV) (Barker et al., 1958; Borman, 1982) is another technique used, which was first introduced by Barker et al. The major advantage of SWV over DPV is its speed and higher current compared to analogous-differential pulse response. Several other electrochemical techniques include potentiometry (Joseph, 2000), which can provide information about the analyte using the potential between the two electrodes; Bulk electrolysis can be used for bulk synthesis of product, which requires large ration of surface area to solution volume.

## 3 Functionalization

### 3.1 C-N functionalization

Electrochemical reactions are important methods for C-H functionalization in a wide range of heterocyclic substrates. They have been proven to be very effective for substrates that are resistant to the conventional method of reaction initiation by peroxide radicals. It is important for us to study and understand the mechanistic approach of electrochemical C-H functionalization because it is of great interest in medicinal chemistry.

Electrochemical oxidation is an important method to functionalize the C-H bonds of aromatic and benzylic carbons involving single-electron transfer (SET). However, the limitation of the method lies in the fact that the aromatic and benzylic compounds in the presence of unprotected imidazole get electrochemically oxidized. There have been numerous reviews in this field. However, the ones by Zheng et al. (2020) and Kärkäs (2018) stand out. In this regard, work has been done by the Yoshida group wherein a new approach for C-N coupling of protected imidazoles was conducted based on the electrooxidative C-H functionalization of organic counterparts. This approach is applicable to aromatic and benzylic compounds. The group has demonstrated the electrooxidative C-N coupling of substrates such as N-methylimidazole and its aromatic and benzylic derivatives. The reaction proceeds with the formation of an electrochemically inactive imidazolium ion, which is transformed into N-benzylimidazoles or N-aryl with piperidine treatment. The reaction is highly chemoselective and straightforward and does not involve metal catalysts in the synthesis of N-substituted imidazoles (Morofuji et al., 2014).

**FIGURE 4:**
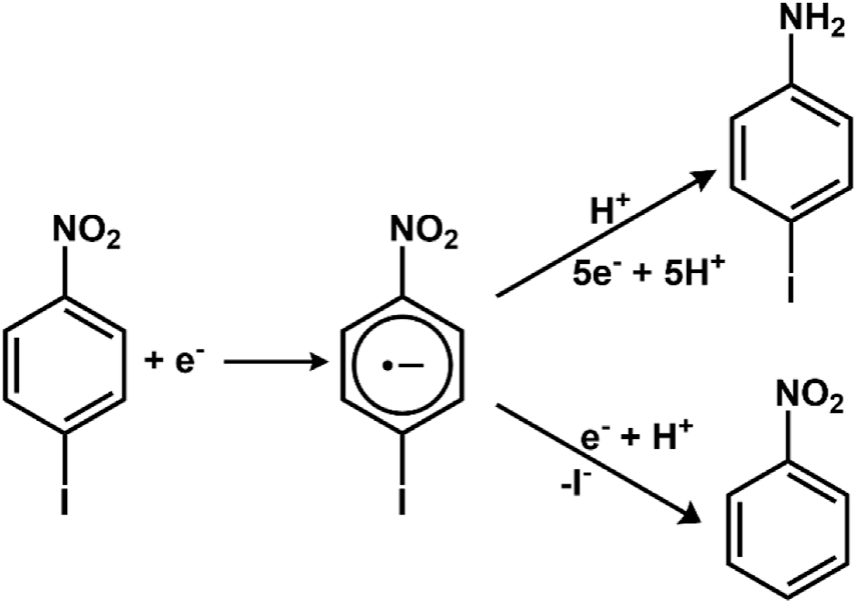
Different reaction pathways under coupled chemical process.

The group also pioneered in developing several innovative methods for C-H amination, including metal-free benzylic amination *via* electrochemically generated benzylaminosulfonium ion (Hayashi et al., 2017). One of the methods introduces the intermediacy of electrooxidatively inactive cationic intermediates, which limits overoxidation. In order to address the problem of regioselectivity in some cases, an intermolecular approach was adopted. An organic transformation was designed using a pyridine ring instead of a pyrimidine ring, and 2-pyrimidyloxybenzene was synthesized using 2-bromopyrimidine and phenol. The oxidation of 2-pyrimidyloxybenzene at an anode generated a cyclized cationic intermediate. Furthermore, on treatment with piperidine, it gave 2-aminobenzoxazole, which is an important therapeutic molecule (Morofuji et al., 2015). One of the significant examples of C-H amination (Figure 4) was reported by the Mei group. They had worked on C-H amination of arenes with secondary amines electrochemically using Cu(OTf)_2_. *n*-Bu_4_NI was used as a redox mediator. The analysis of cyclic voltammogram, radical inhibition experiments, kinetic profiles, and isotopic effects are indicative of the fact that the reaction proceeds through an SET reaction, and it has been assumed that a Cu (III) species might be involved (Yang et al., 2018). The Ackermann group worked on electrochemical C-H amination of azole through cross-dehydrogenative N-H/C-H functionalization. The reaction could proceed easily without the involvement of a metal catalyst or any additional electrolyte. Instead, the acid additive was found to assist the electrochemical C-H amination (Sauermann et al., 2018). The group has also reported C-H amination *via* cobalt-catalyzed C-H Activation. During cyclic voltammetric studies, the oxidative potential of the cobalt catalyst (1.05 V_SCE_) in the presence of a base, KOAc, supported the idea of SET. In fact, a higher potential of 1.51 V_SCE_ indicated the generation of Co (III) species during SET (Sauermann et al., 2018) using SCE as a reference electrode. The group also has similar interesting works in this regard (Tian et al., 2018).

One of the noteworthy works on C-H amination was from the Aiwen Lei group, wherein they reported a simple method for the synthesis of N-aryl phenothiazines from phenothiazines and unprotected phenols. The reaction was performed in a CH_3_CN/CH_3_OH solvent in an undivided cell under a constant current of 7 mA for 100 min, and p-methoxyphenol reacted with phenothiazine in the presence of ^n^Bu_4_NBF_4_ as the electrolyte (Figure 3). The mechanistic studies have highlighted the fact that amine substrates get oxidized to radical cation and lead to the initiation of the reaction (Tang et al., 2018b). The group developed an approach for oxidative intramolecular C(sp^3^)-H amination of amides electrochemically in an undivided cell using platinum as a cathode and a carbon rod as an anode under constant current. While tetrabutylammonium acetate was incorporated as an electrolyte, it was also found to promote cleavage of the N-H bond and initiate hydrogen bond formation with amide. In fact, the intermolecular inert δ C(sp^3^)-H amination was found to occur through the 1,5-HAT process (Hu et al., 2018b). In 2019, the Lei group further reported intermolecular cross-coupling between aromatic substrates and sulphonimides (aromatic C-H imidation) induced by electrochemical oxidation (Hu et al., 2019). The reaction follows an N-radical addition pathway, and the cyclic voltammetry experiments showed that the N-centered imidyl radicals are produced as a result of proton-coupled electron transfer (PCET), which is mediated by anodic oxidation and ^n^Bu_4_NOAc (tetrabutylammonium acetate). The group also reported electrochemical dehydrogenative C(sp^3^)-H/N-H cross-coupling for developing a series of N-Mannich bases, wherein controlled experiments indicated that acetic acid played a major role in the formation of C(sp^3^)-N bond (Wang et al., 2019b).

Another work on oxidative C(sp^3^)-H/N-H cross-coupling of imidazopyridines with diarylamines was reported by the same group (Liu et al., 2019). There have been numerous reports on dehydrogenative C(sp^3^)-H/N-H cross-coupling (Wang and Stahl, 2019). The Lin group reported electrochemical diazidation of alkenes using an Mn-based catalyst (Fu et al., 2017). The group converted alkenes in the presence of sodium azide to 1,2-diazides. Further, the use of Mn-based catalysts and electrochemical energy under mild conditions of 1,2-diazides was reduced to vicinal diamines, which are important precursors for the natural product and molecular catalyst and as therapeutic agents. The formation of the second C-N bond is very challenging. Hence, to eliminate the problem, a redox active catalyst was introduced in the system through kinetic control to generate selectivity. Thus, the catalyst could very easily react with N_3_^−^ to form a complex and ultimately form a metal adduct (M-N_3_), which would react with *in situ* generated carbon radical *via* oxidative addition or direct group transfer before reductive elimination to finish the cycle of diazidation.

The role of hydrogen atom transfer during electrochemical dehydrogenative C(sp^3^)-H amination was highlighted beautifully by the Magnus Rueping group (Nikolaienko et al., 2019). The electrooxidative amination of C(sp^2^)-H bonds was also reported by the group of Ian A. Nicholls, which involves cross-coupling of amines with aryl amides using Cu(OAc)_2_ as a catalyst due to the synergy between Cu catalyst and the electrocatalysis under mild conditions (Kathiravan et al., 2019). The Li-Zhu Wu group reported electrochemical ortho-amination of alkoxyl arene facilitated by trifluoroacetic acid. Trifluoroacetic acid, in this case, regulates the regioselectivity. The control experiments indicate that the arene radical cation intermediate is involved in the successful conversion. Increasing the current density during the experiments preserves the selectivity but decreases the yield (Wang et al., 2019a).

Another interesting work on the electrochemical oxidation of amino acids was reported by the Yahui Wang group. The group reported a decarboxylative C(sp^3^)-N coupling reaction that proceeded *via* anodic oxidative decarboxylation of carboxylic acid to form stabilized carbocations, which are trapped by amides or azoles to form C-N bonds. N-based nucleophiles need to be reactive and stable under electrolytic conditions to trap the stable iminium cations generated from the anodic oxidation of amino acids. Azoles, in this case, fulfill both criteria (Shao et al., 2019). Another attempt toward electrochemical oxidative C-N bond formation *via* C_sp_^2^-C _sp_^3^ bond cleavage was reported by the Zeng group and the Jiao group (Adeli et al., 2019).

### 3.2 C-C coupling

The Magnus Rueping group reported the first electrochemical approach for cross-electrophile coupling to generate 1,1-diarylalkane derivatives from alkyl and aryl halides using a nickel-based catalyst, (6,6′-dimethyl-2,2′-dipyridyl)NiBr_2_. The mechanistic studies reveal the generation of Ni^0^ species. Further, the Ni hydride species produced was found to facilitate the chain walking pathway to efficiently catalyze the electro-reductive process and hydroarylation (Kumar et al., 2020). The Antonchick group nicely summarized important works reported in the field of metal-free oxidative C-C bond formation through C-H bond functionalization (Narayan et al., 2015). The Waldvogel group had several methods for achieving homocoupling in phenolic substrates (Kirste et al., 2011a; Kirste et al., 2011b). In this regard, the group developed an active molybdenum-based electrode anode for performing dehydrogenative aryl coupling. This electrode in HFIP formed a high valent molybdenum species, which formed an active surface layer upon dissolving in the electrolyte (Beil et al., 2018).

The radical-cation-pool strategy could be used to synthesize asymmetrical biaryls from inactivated electron-rich compounds. Its origin could be traced back to the cation-pool method (Okajima et al., 2005; Nokami et al., 2008; Morofuji et al., 2012). The Yoshida group exploited the method to report C-C bond formation *via* C-H cross-coupling of two inactivated aromatic compounds. The process could be traced back to the generation of aromatic radical cations under an oxidative environment, followed by the coupling of the radical cation species with other aromatic substrates under a non-oxidative environment. The Waldvogel group reported metal-free electrochemical cross-coupling of phenols and arenes in fluorinated solvent using boron-doped diamond (BDE) anodes (Kirste et al., 2012). The group is also credited with electrochemical dehydrogenative cross-coupling to produce a series of 2,2′-diaminobiaryls (Schulz et al., 2017) and C-C cross-coupling of thiophenes with phenols (Wiebe et al., 2017). Cross-dehydrogenative coupling (Li, 2009) is an important strategy for the construction of C-C bond formation by activating two different C-H bonds. The classical method involves the use of a stoichiometric amount of oxidants to remove hydrogen and electrons, leading to the generation of intermediates that activate the C-H bond. However, the presence of a stoichiometric amount of oxidant would limit the application. In recent years, the focus has shifted toward a \ greener approach (Hu et al., 2018a).

### 3.3 C-H oxygenation

The Baran and Blackmond group reported an electrochemical initiation method, which generated increased yields during the C-H activation of complex pharmaceutical substrates (O’Brien et al., 2014). Usually, sulfinate radical sources are used to synthesize complex alkyl- and fluoroalkyl-substituted heterocycles. However, they display a low yield for a large number of substrates. The electrochemical C-H functionalization of carbamates using N-acyliminium ion is a very important method for synthesizing biologically important precursors, but the mechanism was not understood. One of the important works by the Baran group involves the activation of the inactivated C-H group through electrochemical oxidation *via* simple carbon and nickel electrodes and quinuclidine as a redox mediator. The electrochemical oxidation of sclareolide at C2 occurred at a lower potential owing to the presence of quinuclidine. Hence, the units, silyl ether, and ketones, among others, were well tolerated. The mechanism involves the formation of quinuclidine cation at the anode. It cleaves the inactivated C-H bond, followed by oxidation using molecular oxygen. The Baran group also reported phenomenal work on electrochemical allylic C-H oxidation (Horn et al., 2016). The allylic oxidation suffers from the use of toxic reagents, low yield, and expensive catalysts. The situation could be modified by introducing a co-oxidant, identifying a more effective electrochemical mediator, and designing an inexpensive reaction setup. Cl_4_NHPI was chosen as the electrochemical mediator instead of the conventional NHPI because it was found that attaching an electron-withdrawing group to the phthalimide moiety increased the activity of the catalyst. The mechanistic cycle of allylic oxidation was explained as follows: the pyridine deprotonated the Cl_4_NHPI followed by anodic oxidation, leading to the generation of tetra-chlorophthalimido N-oxyl radical species. The olefinic substrate would undergo hydrogen atom abstraction regenerating a stable allylic radical and Cl_4_NHPI. The ^t^BuOO^**.**^ radical would form allylic peroxide, which generates enone on the elimination of ^t^BuOH.

Another work on electrochemical allylic oxidation was reported by the Waldvogel group (Edinger and Waldvogel, 2014), wherein they have further worked on the innovation introduced by Baran et al. The molecular oxygen as a co-oxidant was replaced by tert-butylhydroperoxide. The reactivity of the mediator was enhanced by substituting the tetrachloro-derivative. High-surface glassy carbon was used to lower the current density at the anode (Waldvogel and Selt, 2016). Another work by Baran involves C-H oxidation, wherein quinuclidine radical cation was generated through anodic oxidation, which led to the cleavage of the inactivated C-H bond and molecular oxygen producing the oxidation product. HFIP served as an electron acceptor leading to the generation of H_2_ during the cathodic process (Kawamata et al., 2017). The group has also worked significantly in the field of electrochemical oxidative dimerization (Rosen et al., 2014).

The Liu group reported electrochemical oxidation of the benzylic C-H bond. The reaction was performed at room temperature and in aqueous solvents. The anodic oxidation of tBuOOH resulted in the formation of ^t^BuOO^**.**^ radical, which leads to C-H abstraction at the benzylic position of the substrate, resulting in the formation of benzyl radical. This benzylic radical reacts with tBuOOH, generating corresponding oxygenated products. The KIE experiments suggested the $k_{H}/k_{D}$ value close to 19.1, indicating that C-H abstraction is the rate-limiting step (Marko et al., 2019). The Stahl group worked on the electrochemical oxygenation of the benzylic C-H bond and dehydrogenation of alcohols to ketones catalyzed by the tetraamido macrocyclic ligand (TAML) Fe-oxo species. The generation of Fe^IV^ and Fe^V^ at increasing potentials (800 and 1,250 Mv) was determined through voltammetric analysis, and their sustained electrolysis led to the formation of desired products (Das et al., 2019).

**FIGURE 5:**
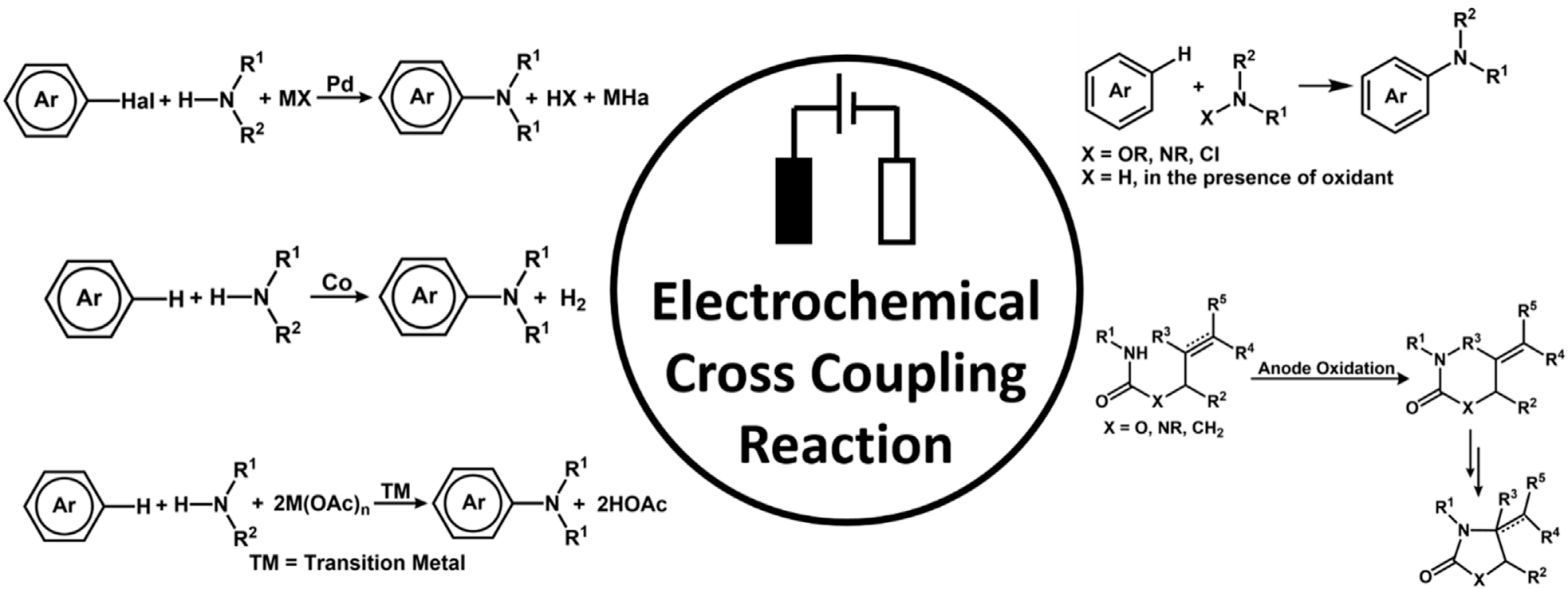
Strategies for different electrochemical cross-coupling, oxidative amination, C-N bond formation, and C-H imidation.

The Aiwen Lei group reported C-H oxygenation at ambient conditions at 23°C catalyzed by cobalt catalyst. The reaction proceeded *via* the oxidation of substrate by a cobalt (III) species generated by a mixture of catalyst Co(oAc)_2_ and NaOPiv in MeOH (Sauermann et al., 2017). An important work on C(sp^2^)-H acetoxylation of arenes *via* electrochemical oxidation was reported by the Zhang group (Li et al., 2017). The reaction does not employ any stoichiometric amount of oxidant and occurs at a mild temperature. The kinetic isotopic effect gave a mechanistic insight, which suggests Pd(II) atom coordinated with the N-atom of the oxime. It is followed by the rate-determining step of C-H activation and further undergoes N oxidation of Pd(II) to Pd(IV) and reductive elimination to give the desired product. The Little group worked on electrochemically *in situ* generated tris(p-bromophenyl) aminium radical cation initiated aromatic C-H bond functionalization. The solvents play a very important role in C-H Activation (Figure 5), protecting the product and generating highly active catalysts and, even sometimes, the air-regenerable oxidant (Hashiguchi et al., 2012).

An effective electrochemical Friedel–Crafts alkylation protocol was developed for electron-rich aromatic substituents with N-vinyl amides (Li et al., 2015). The *in situ*-generated TBPA cation was used as an electron transfer reagent for initiating a cationic chain reaction. Polymeric ionic liquid-carbon black composite was used as a supporting electrolyte. Cyclic voltammetry and control experiments proved that cationic chain reaction proceeds through oxidative activation of the C-H bonds of aromatic compounds. The group also successfully achieved electrochemical aziridination of alkenes (Chen et al., 2015a). A number of structural variants of aziridines were synthesized using an undivided cell operating at a constant current and were mediated by n-Bu_4_NI in catalytic amounts. The reaction followed a radical pathway and occurred without the absence of additional conducting salt. The Xu group also reported electrochemically driven decarboxylative C-H functionalization of heteroarenes. The work dealt with C-H alkylation and carbamoylation of electron-deficient N-heterocycles with oxamic acid and carboxylic acid through H_2_ evolution (Lai et al., 2020).

An interesting piece of work was reported by the Ding group on direct and selective electrochemical conversion of benzyl C-H using a sole oxygen source and water as a co-solvent (Sun et al., 2020). The on-set potential of benzyl C-H bond activation could be reduced significantly due to the addition of water, which was enhanced further by oxygen vacancy-rich MnO_2_ as an electrocatalyst. The approach excluded the use of mediators. In recent studies, the focus has been more on finding green sources and overcoming anodic potential. The above section demonstrated how the electro-oxidation of the C-H bond gave rise to either carbon-centered cations or radicals, which upon the attack by oxygen-containing reagents, could offer myriad products, such as aldehydes, ketones, and esters with C-O bond formation. The electrochemical approach could be used to manipulate the oxidation state of the product by offering precise anodic potential, thus providing control over the selectivity of oxidation.

### 3.4 Sulphonation, selenation, and silylation

The Aiwen Lei group worked on direct arylsulfonylation of ynones with sulfinic acid through an electrooxidative pathway. The reaction was performed in an undivided cell using two platinum plates as a cathode and working electrode, TBAI as redox catalyst, CH_3_CN/DCE as a solvent, and LiClO_4_ as electrolytes while passing a constant current of 10 mA·cm^−2^. The reaction produced a variety of indenones in excellent yield. The group also developed a selective and highly efficient protocol for the synthesis of β-alkoxy and β-amino sulfides *via* electrochemical oxidative aminosulfenylation and oxysulfenylation of alkenes involving thiophenols/thiols as thiolating agents (Yuan et al., 2018).

Interesting work was reported by the Sun group on direct C(sp^2^)-H selenation of indoles without using an oxidant- and transition metal-free method. The control experiments led to the following observation: atmospheric air pressure led to a higher rate of selenation, protonic solvent interfered slightly with the yield, and the addition of iodine salt to the system enhanced the selenation process. Hence, KI was introduced into the system as a redox mediator and an electrolyte to increase the conductivity. The indoles and their substituents underwent C-H selenation electrocatalytically at the C3 position and generated selenyl indoles at a good yield (Zhang et al., 2018b).

**FIGURE 6:**
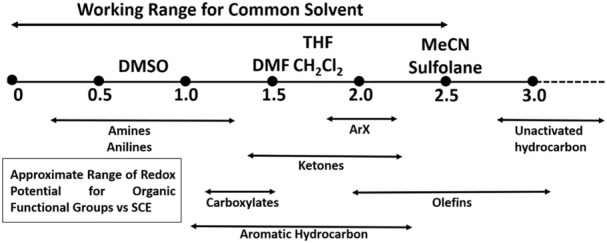
The challenge of electrochemical C-H activation.

An interesting piece by Huang reported electrochemically driven stereoselective synthesis of sulfur constituting β-enaminonitrile derivatives through oxidative C(sp^3^)-H functionalization of acetonitrile (He et al., 2019). The stereoselectivity was enhanced and used in the presence of a phosphine oxide catalyst. The activation of the C(sp^3^)-H bond produces cyanomethyl radicals in the presence of KI as a redox catalyst at a lower anodic potential leading to the formation of the C-S bond and enamines. Another example of C-S bond formation was reported by the Yi Pan group. The work was based on the Ullmann type of thiolation of aryl iodides catalyzed by NiCl_2_.glyme (Figure 6). The reaction was performed in an undivided cell in the presence of graphene/nickel foam electrodes because they help in enhancing the charge exchange. However, the reaction observed an increased yield due to the ratio of added electrolytes. The highest isolated yield was observed with the addition of three equivalent LiBr at E_cell_ of 3 V (Wang et al., 2019c).

The Huo Cao group reported electrochemical diselenylation of indolizines using selenide, ketones, and pyridines in an external oxidant and transition metal catalyst-free condition in an undivided cell. The work dealt extensively with electrochemically driven intermolecular C-Se formation of α-bromoketones, 2-methylpyridines with diselenides producing diselenylated indolizines through direct C-H bond selenylation. A series of electrolytes were tested, such as KI, KBr, n-Bu_4_NBF_4_, n-Bu_4_NI, and LiClO_4_, and it was observed that halogen anions played a key role in the transformation. Subsequently, KI was chosen because it generated the highest yield (Li et al., 2020). An approach toward direct trifluoromethylsilylation and cyanosilylation of aldehydes *via* an electrochemically induced intramolecular pathway was reported by the Kedong Yuan group (Yang et al., 2020). The cathodic activation of trimethylsilyl reagents, such as SiMe_3_CF_3_ and SiMe_3_CN, allows the Si-O affinity to generate concerted anion intermediates, which further generates products with higher selectivity and yield *via* intramolecular -CF_3_ and -CN migration. The generation of concerted anion intermediate through direct cathodic activation of CF_3_-SiMe_3_ was energetically favorable (ΔG = −0.29 V) from DFT calculations.

### 3.5 Halogenation and trifluoromethylation

**FIGURE 7:**
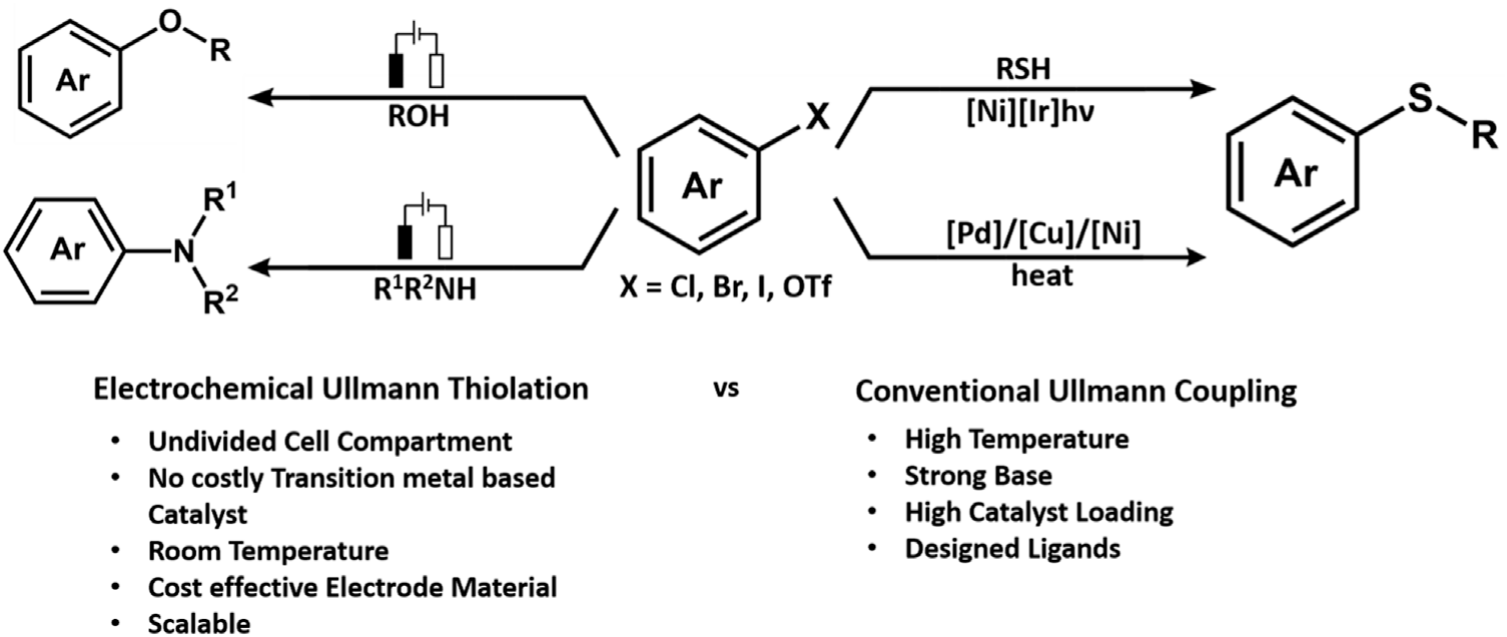
Schematic comparing electrochemical Ullmann thiolation with conventional Ullmann coupling.

The Zeng group reported electrochemical Minisci-type trifluoromethylation of electron-deficient heterocycles wherein bromide ions are used as redox mediators (Dou et al., 2019). The efficiency of the process is maintained due to *in situ* formation of sulphonyl hypobromite intermediate, which regulates the concentration of the CF_3_ radical. The reaction proceeds in an undivided cell under galvanostatic conditions without the presence of supporting electrolytes. Another important work on electrochemical decarboxylative trifluoromethylation was reported by the Yubin Huang group (Hong et al., 2019). The group successfully performed decarboxylative trifluoromethylation of α,β-unsaturated carboxylic acid using Langlois reagent, which acts as a trifluoromethyl precursor. The reaction produces C_vinyl_-CF_3_ compounds in good yield. The CF_3_ radical is generated at the anode by CF_3_SO_2_Na *via* SET while releasing SO_2_. The CF_3_ radical then attacks α,β-unsaturated carboxylic acid, which is oxidized to release oxygenated product and CO_2_. Over the years, several electrochemical approaches have been resorted to perform trifluoromethylation (Figure 7). Similarly, the Mo group devised an electrochemical strategy for trifluoromethylation using the Langlois reagent acting as a source of CF_3._ A simple Mn salt, MnBr_2_.4H_2_O, was used as a redox mediator, which is inclusive to a range of functionalized heterocycles, thus yielding a good amount of functionalized heterocycles bearing the CF_3_ moiety. The controlled experiments and mechanistic studies suggest the fact that there is a formation of CF_3_-bound high oxidation state Mn species, which assists in transition-metal-mediated CF_3_ transfer mechanism for C(sp^2^)-H functionalization. The electrochemical reaction between Mn^II^ and CF_3_SO_2_Na was studied using ^19^F NMR Spectroscopy. CF_3_SO_2_Na is known to display a singlet peak at −87.5 ppm, which disappeared on the application of the 1.1 F/mol current. Instead, a new peak appeared at −73.0 ppm, denoting that it was completely consumed on electrolysis generating a new CF_3_ species (Zhang et al., 2019).

An electrochemical approach toward intramolecular oxytrifluoromethylation of N-tethered alkenyl alcohol to generate CF_3_-containing morpholine derivative was reported by the Geraldine Masson group (Claraz et al., 2020). The approach includes direct anodic oxidation using the Langlois reagent as the trifluoromethylating reagent. The desired derivatives of morpholines were achieved by performing cyclization of alken-6-ol with the Langlois reagent using graphitic carbon anode and platinum cathode under constant current in the presence of LiClO_4_ as an electrolyte and a mixture of CH_3_CN and H_2_O as a solvent. The Lei group developed a protocol to perform oxytrifluoromethylation and aminotrifluoromethylation of alkenes under constant current electrolysis under the influence of the Lewis acid catalyst, Y(OTf)_3_. The method ensured the difunctionalization of the C-C bond (Zhang et al., 2018a). In this regard, the Ackermann group reported electrochemical di-/trifluoromethylation of N-substituted acrylamides under catalyst and oxidant-free conditions. The reaction condition displays high tolerance toward the functional group and provides judicious access to 3,4-dihydroquinolin-2(1H)-ones and fluoroalkylated oxindoles. It was observed that with the addition of BHT (7,2,4-di-tert-butyl-4-methylphenol), a radical scavenger to the medium, the trifluoromethylation got completely suppressed, which suggests that SET is involved in the mechanistic process.

Another work reported by the group involves di-/trifluoromethylation or cyclization of N-acrylamides under oxidant-free conditions. The noteworthy features of the work involve 1) effective fluoroalkylation without any involvement of metal catalyst or photoredox conditions; 2) catalyst-free, direct generation of radicals through electrolysis of sulfinate salts; 3) unprecedented mild conditions at 23°C; and 4) abundant scope for the formation of di- and trifluoromethylated oxindoles and 3,4-dihydroquinolin-2(1H)-ones (Ruan et al., 2019). The Yi Pan group reported electrochemical difunctionalization of olefin through the radical fluoroalkylation pathway. The method is advantageous because olefins can be modified easily *via* cascade dual anodic oxidations. The redox potential for the three fluoroalkylating agents—NaSO_2_CF_2_H, NaSO_2_CF_3_, and NaSO_2_C_6_F_13_—was 0.590, 0.814, and 0.742 V, respectively, using cyclic voltammetry. Thus, NaSO_2_CF_2_H can be easily oxidized at the anode. Thus, the order of reactivity of fluoroalkane sulphonates for oxidative radical fluoroalkylation was NaSO_2_CF_3_ > NaSO_2_C_6_F_13_ > NaSO_2_CF_2_H (Zou et al., 2019).

The Yulai Hu group reported electrochemical radical chlorination, bromination, and formyloxylation of various alkenes using NaCl, NaBr, and NaSO_2_CF_3_ as radical sources. (Sun et al., 2019). Electrochemical deconstructive chlorination of cycloalkanols catalyzed by MnCl_2_.4H_2_O was reported by the Morrill group. The reaction proceeded with the formation of alkoxy radical intermediates, leading to the formation of β- and γ-chlorinated ketones (Allen et al., 2019). The Shenlin Huang group was the first to develop an electrochemical approach toward oxydihalogenation of alkynes. The mechanistic scheme involved the generation of Cl^−^ from CHCl_3_ through nucleophilic substitution of I^−^. Furthermore, the Cl^−^ underwent oxidation at the anode to form chlorine radicals, which in addition to the alkyne, produced vinyl radicals. The vinyl radical on oxidation followed by nucleophilic addition of water generated enol moiety. Further enol on recombination with chlorine radical generated α,α-dihaloketone (Meng et al., 2020).

### 3.6 Annulation reaction

**FIGURE 8:**
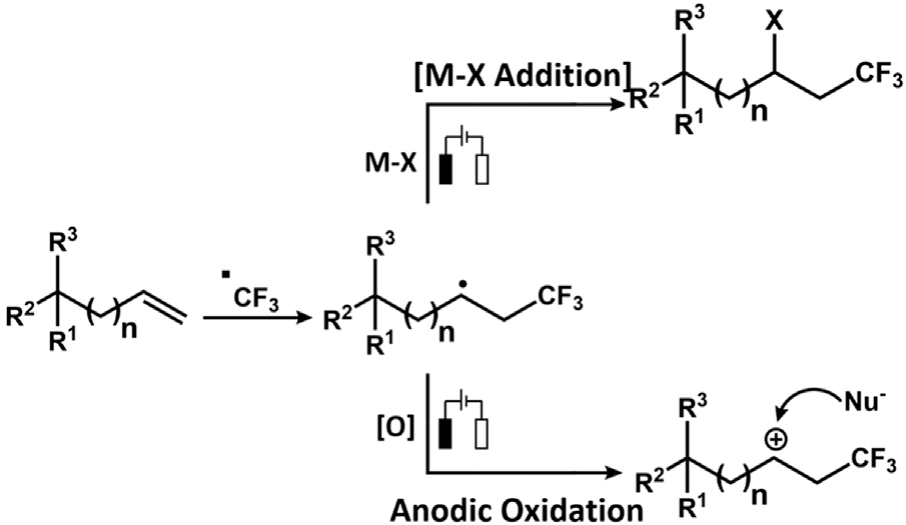
Electrochemical approaches for the generation of Trifluoromethyl radical.

We will briefly discuss the annulation reaction wherein two bonds are generated in a single step. This reaction is an effective way to prepare cyclic compounds (Figure 8). The focus is particularly on dehydrogenative annulation reaction through X-H (heteroatom or X = C) functionalization. The Hai-Chao Xu group demonstrated, for the first time, the synthesis of saturated heterocycles using 1,4-diheteroatom *via* alkene annulations. The annulations of 1,1-diphenylethylene and ethylene glycol were performed in an undivided cell involving a platinum plate as cathode and RVC as anode wherein ^i^PrCO_2_H was used as an acid additive and (2,4-Br_2_C_6_H_3_)_3_N (triarylamine) as redox catalyst (E_p/2_ = 1.48 V *vs*. SCE). The 1,4-dioxane product displayed the highest yield of 91% (Cai and Xu, 2018).

The Aiwen Lei group also contributed significantly to the field of annulation reaction listed in Table 1. In 2018, the group reported electrochemical dehydrogenative C-H/N-H [4 + 2] annulations of aryl vinyl amide with alkene/alkyne using Co(acac)_2_ as a catalyst. The reaction exhibited high functional group tolerance. The controlled experiments for mechanistic studies determined that the key step for the process might be the electrochemical oxidation of the Co(II) complex at the anode (Tang et al., 2018a). The Ackermann group reported for the first time an electrochemical alkyne annulation reaction *via* C-H/N-H activation catalyzed by a cobalt-based catalyst, Co(OAc)_2._ The cyclic voltammetry experiments for C-H activation revealed that the oxidation potential of the catalyst (1.19 V_SCE_) was lower by 320 mV than that of the substrates (1.51 V_SCE_) using SCE as the reference electrode, indicating that the catalyst promotes SET. The group also reported for the first time the metalla-electrocatalyzed C-H activation through Rhoda-catalyzed alkyne annulations. A modular electro-flow cell was incorporated with porous graphite felt, which ensured efficient turnover number, leading to successful C-H/N-H activation with excellent regioselectivity and chemoselectivity. The catalyst Rh(III) carboxylate was regenerated *via* anodic oxidation, and H_2_ was the sole byproduct of cathodic reduction (Kong et al., 2019). The group also reported the electrochemical synthesis of aza-polycyclic aromatic hydrocarbon through rhodium-catalyzed cascade C-H activation and alkyne annulations (Kong et al., 2020). A multifunctional O-methylamidoxime was designed to sustain high regio- and chemoselectivity. The reaction was performed using [ $C_{p}^{*}$ RhCl_2_]_2_ as the catalyst precursor and MeOH as the solvent, and KOAc was used as a base under a constant current of 4.0 mA. In fact, the reaction generated a higher yield in a shorter span, indicating that a prolonged duration of electrolysis can lead to the generation of undesired side products. The Xu group reported an electrochemically driven dehydrogenative annulation reaction between anilines and alkynes for the synthesis of indoles using ruthenium catalyst, [RuCl_2_(p-cymene)]_2_ (Xu et al., 2018). The Mei group did notable work in the field of electrochemically driven vinylic C-H functionalization of acrylic acid with alkynes catalyzed by $\left(C_{p}^{*}\right.$ IrCl_2_)_2._ The Ir-based catalyst is effective in synthesizing α-pyrones in an excellent yield. The mechanistic studies herein provide the insight that anodic oxidation is a crucial process for product formation and the regeneration of Ir(III) intermediate from a diene-Ir(I) complex that is coordinatively saturated 18e^−^ complex (Yang et al., 2019).

### 3.7 Synthesis of cyclic carbamate

The Stahl group reported an electrochemical method for α-oxygenation of cyclic carbamates using water as a nucleophile, and bicyclic aminoxyl was used as a mediator. In the aminoxyl-mediated electrochemical process, the substrate oxygenation is observed to proceed at a potential lower by 1 V compared to the redox potential of the carbamate substrate, measured using an Ag/AgNO_3_ reference electrode, because we observe functional group compatibility, which is inaccessible in the case of conventional Shono-type reaction, which proceeds *via* direct electrochemical substrate oxidation. This was, in fact, one of the first examples of α-functionalization of non-activated cyclic carbamates using oxoammonium oxidants (Wang et al., 2018). The Urieta group performed electrochemical studies on the α-alkoxylation of carbamates, which led to the conclusion that it followed an ECEC mechanism, wherein the first two steps occur in a concerted manner. This was further confirmed by theoretical studies on absolute redox potential for stepwise and concerted pathways. The reaction shows anomalous regioselectivity, which was caused by the dipole moment vector of the asymmetric carbamates, which, in interaction with the positive charge of the anode, orients the molecule in a way that it favors the cleavage of the C-H bond for the less substituted site.

### 3.8 C-H functionalization of ethers and esters

A very important piece of work was reported by the Tristan H. Lambert group that reported C-H functionalization of ethers catalyzed by trisaminocyclopropenium (TAC) at a mild electrochemical potential under visible light irradiation (Huang et al., 2020). The reaction occurs under oxidant-free conditions in which ethers undergo coupling reaction with substrates such as purines, pyrazoles, alkynes, and alkenes with high regioselectivity toward less hindered α-position. The reaction proceeds *via* hydrogen atom transfer reaction from the substrates to the photoexcited TAC radical dication. The studies show a new direction toward combining photocatalysis with electrocatalysis. The Waldvogel group reported work on the electrochemically driven synthesis of 1,1,1,3,3,3- hexafluoroisopropanol aryl ether in the presence of BDE electrodes. A base such as triethylamine was used because it formed a highly conductive media with HFIP, resulting in the superfluous behavior of the additional electrolyte (Waldvogel et al., 2020) see in Table 1. The mechanistic study suggests that the reaction might proceed *via* ECEC-type mechanistic.

### 3.9 Alcohol oxidation

Recently, the focus has shifted to alcohol oxidations in fuel cells. For low-temperature fuel cells, methanol could be an alternative to hydrogen because methanol could easily be oxidized to CO_2_ without having to break the C-C bond. Organic aminoxyls, in the presence of chemical oxidants such as Br_2_, NaOCl, and PhI(OAc)_2_, act as widely accepted mediators for the transformation in both acidic and basic conditions (Tojo and Fernández, 2010b). Electrochemical alcohol oxidation mediated by aminoxyl was documented extensively in different comprehensive reviews (Tojo and Fernández, 2010a; Nutting et al., 2018). The Stahl group reported electrochemical alcohol oxidation using aminoxyl mediators, including TEMPO, its derivatives, and several bicyclic aminoxyl species, such as ABNO. 4-PhCO_2_-TEMPO and 4-AcNH-TEMPO (ACT) were observed to have the highest E_1/2_ leading to the conclusion that the driving force for the transformation is more powerful than the steric effect. In fact, this behavior contradicts the observation for the reaction catalyzed by organic aminoxyls in the presence of chemical oxidants such as Br_2_, NaOCl, and PhI(OAc)_2_, wherein sterically less hindered bicyclic aminoxyls prove to be an effective catalyst (Rafiee et al., 2015; Cardiel et al., 2019). Over the years, ACT has proven to be an effective electrocatalytic mediator for alcohol oxidation with broader substrate scope. ACT was reported to be effective at selective alcohol oxidation in lignin (Cardiel et al., 2019). However, their efficiency is limited by the diffusion of the catalyst to the surface of the electrode. The issue could be addressed by immobilizing the catalyst to the surface of the electrode.

Pyrene-TEMPO conjugate catalysts adsorbed on MWCNTs were far more superlative compared to ACT (Das and Stahl, 2017). The oxidation reaction of 4-methoxybenzyl alcohol had a TOF of approximately 4,000 h^−1^ and TON >1,800 (Rafiee et al., 2018). Cu-based aminoxyl systems were highly effective for the aerobic oxidation of alcohol with high functional group tolerance (Steves and Stahl, 2013; Ryland and Stahl, 2014). While aminoxyl-driven alcohol oxidation employing chemical oxidants was two-electron processes, the mechanistic studies have validated that the aminoxyl species and Cu^II^ serve as a one-electron oxidant (Hoover et al., 2013; Ryland et al., 2014; McCann and Stahl, 2015). The electrochemical alcohol oxidation using the Cu/TEMPO system operated at 0.5 V lower potential than the reaction based on only TEMPO (Badalyan and Stahl, 2016; Ryan et al., 2019).

In a report by the Whitesides group, Fe^2+^/Fe^3+^ redox couple was used as a mediator for methane oxidation with Pt black catalyst (Bergens et al., 1994). The Fe^2+^/Fe^3+^ as a redox couple mediator was also extended to ethylene glycol, peat, kraft lignin, and sub-bituminous coal as fuel without additional catalysts (Gorman et al., 1996; Weibel et al., 2005). These mediators could function using unconventional fuels. Polyoxometalates have been widely used as catalysts in the oxidation of alcohols, hydrogen, and biomass in fuel cells. In a report by the Deng group, H_3_PMo_12_O_40_ and polyol were irradiated with a metal halide lamp for 17.5 h, after which the solution was passed into an anodic compartment that employed H_12_P_3_Mo_18_V_7_O_85_ as the cathodic POM mediator, leading to enhanced power densities (Wu et al., 2016b). Sulphonated anthraquinone was also tested as a mediator on different kinds of fuel, such as alcohol, carbohydrates, and lignin (Hertl and Weetall, 1985b; Weetal et al., 1985). Thus, mediated electrochemistry provides enough opportunity for power generation in complex fuels. Low-potential polyoxometalates proved to be good anodic mediators with greater redox reversibility, stability, and low cost. The mediated cells, which use biomass, alcohol, and other unconventional fuels, produce lower power output than conventional fuel cells. However, the ability to replace the Pt and non-Pt group metal by deriving power through unconventional sources would further lead to new developments.

## 4 Electrochemical CO_2_ reduction

A selective and efficient method for electrochemical carbon dioxide reduction (Lu et al., 2014b) is one of the greatest challenges in the field of artificial photosynthesis to produce cleaner fuels. In 2014, the Salehi-Khojin group (Shen et al., 2016) reported molybdenum disulfide as a superior catalyst in an ionic medium for carbon dioxide reduction compared to noble metal owing to high current density and low overpotential of 54 mV, as measured against Ag wire as a reference electrode (Asadi et al., 2014). The superior activity of molybdenum disulfide is due to its molybdenum terminated edges, which are responsible for its high d-electron density and metallic character. It has been observed that vertically aligned MoS_2_ displays a high CO_2_ reduction current density of around 130 mA·cm^−2^ for both low- and high-applied potential owing to the presence of a high density of active sites, especially Mo atoms. The group also reported 2D nanoflakes of metal dichalcogenides for catalyzing CO_2_ reduction in an EMIM-BF_4_ medium. The nanoflakes exhibited a current density of 18.95 mA·cm^−2^ and a faradaic efficiency of 24%. The CO formation reported a turnover frequency of 0.28 with an overpotential as low as 54 mV. The Norskov group (Chen et al., 2016b; Shi et al., 2016) also reported molybdenum sulfides and selenides as probable electrocatalysts for CO_2_ reduction (Chan et al., 2014). There was also an investigation on the electrochemical reduction of CO_2_ to CH_4_ *via* eight electron transfer mechanisms using tungsten carbides (WC) and transition metal decorated WC (Wannakao et al., 2015). Silver has always been a traditional electrocatalyst in different kinds of reactions (Ma et al., 2014; Ma et al., 2016b; Singh et al., 2016). The Jiao group nanoporous silver catalyst that reduces CO_2_ to CO with 92% selectivity 3,000 times increased the rate of current compared to its polycrystalline counterpart at a moderate overpotential, which is <0.50 V (Lu et al., 2014a). Such high activity is owing to the greater stability of ${CO}_{2}^{-}$ intermediates on a highly curved surface, thus, requiring smaller overpotentials to overcome the thermodynamic barrier. The selective reduction of CO_2_ to CO is achieved by using Ag nanoparticles supported over carbon. The Ag/C electrodes decreased the overpotential by a range of 300 mV at 1 mA/cm^2^, and a fourfold FE was obtained at −0.75 V *vs.* RHE (Kim et al., 2015).

The Hyungjun Kim group reported the covalency-aided electrochemical reaction (CAER) wherein the p-block dopants affect reaction energy by imposing partial covalency in the metal catalyst that enhances the catalytic activity beyond the modulations arising from d-block dopants (Lim et al., 2014). The Yang group explored the activity of gold-copper bimetallic nanoparticles, which are majorly governed by two factors, namely, the geometric and electronic effects, which help determine their catalytic activities (Kim et al., 2014). The Thomas J. Meyer group (Zhang et al., 2014b) synthesized tin nanocrystals through the hydrothermal method in which selective reduction of CO_2_ to formate occurs at an overpotential as low as 340 mV (Zhang et al., 2014a). The maximum faradaic efficiency for formate formation was calculated as 93%, under a current density of >10%, and high stability on graphene support. The increased reactivity toward carbon dioxide reduction is due to two factors: 1) the strength of interaction between the tin surface and ${CO}_{2}^{.-}$ and 2) the kinetic activation toward protonation followed by reduction. The group also had nitrogen-doped carbon nanotubes (Chai and Guo, 2016) as robust electrocatalysts for the reduction of carbon-reported dioxide to formate in aqueous media wherein polyethylenimine (PEI) acts as a co-catalyst. Another work on nanoporous Tin foam as an electrocatalyst for the conversion of CO_2_ to formate was reported by the TaO group (Du et al., 2016). The catalyst displayed high selectivity toward formate. The reaction proceeded in NaHCO_3_ solution and displayed FE of 90% with a current density of 23 mA·cm^−2^. The electrodes were stable during long-term electrolysis at −2.0 V *vs.* Ag/AgCl. PEI displayed high adsorption capacity and selectivity toward CO_2_. Gold inverse opal thin films (Au-IO) are known for their product selectivity toward CO during CO_2_ reduction. The Sun group reported ultrathin Au nanowires with activated edge sites for selective CO_2_ reduction to CO (Zhu et al., 2014). The reduction is performed using these catalysts at an onset potential of −0.2 V (*vs.* reversible hydrogen electrode) in 0.5 M KHCO_3_ solution. The faradaic efficiency of the process is observed as 94% at a potential of −0.35 V. The LBL assembly of Au nanoparticles on carbon nanotubes showed promising results for the electrochemical reduction of CO_2_ to CO (Huan et al., 2016).

There have also been reports on the electrochemical methanation of carbon dioxide using a dispersible copper nanoscale catalyst. The Alivisatos group demonstrated how copper nanoparticles supported on glassy carbon (n-Cu/C) were almost four times more effective than high-purity copper foil electrodes (Manthiram et al., 2014). The Cu (core)/Cuo (shell) catalyst was reported to be effective for the electrochemical reduction of carbon dioxide to formic acid (Lan et al., 2014). With a catalyst loading of 1.0 mg·cm^−2^, the faradaic efficiency of CO and HCOOH was greater than other catalyst loading. One of the significant works on CO_2_ reduction to C3-C4 products, such as n-propanol and n-butane, was reported by the Jaeyoung Lee group. They had reported a Cl-induced biphasic electrode consisting of (Cu_2_O) and metallic copper (Cu). The synergistic effect between (Cu_2_O) and metallic copper (Cu) led to an abundance of Cu^+1^ and stabilization of the reaction intermediate (Lee et al., 2015). Copper nanoforms with hierarchical porosity have also been used for electrochemical CO_2_ reduction (Sen et al., 2014). The Collins group reported Cu nanoparticles supported on single-walled carbon nanotube (SWCNT), Ketjen black (KB), and carbon black (CB) for effective reduction of CO_2_ to hydrocarbons such as (CH_4_, C_2_H_2_, and C_2_H_4_) (Baturina et al., 2014). The size of Cu nanoparticles ranged from 10 to 30 nm. The supported nanoparticles were less susceptible to aerobic oxidation and agglomeration during the reductive treatment in H_2._ The Feng Jiao group (Rosen et al., 2015b) developed highly dense Cu nanowires (Ma et al., 2016a) for electrochemical CO_2_ reduction. It required a minimal overpotential of 0.3 V to reach a current density of 1 mA/cm^2^. Ag/AgCl was used as a reference electrode. It displayed an FE of ∼60% toward CO production. Another notable work reports Cu nanocubes as electrocatalysts with the greatest selectivity toward ethylene as a product for CO_2_ reduction, wherein the nanocube structure favors the formation of multicarbon products. It was observed that the local pH was much higher than the bulk pH during the reaction, which enhanced the selectivity of the product. The higher roughness of the nanostructured catalyst draws a higher current of −0.5 V (Roberts et al., 2015). There were numerous reports on CO_2_ reduction using copper-based electrocatalysts (Zhang et al., 2014c; Cheng et al., 2016) such as copper meso-/nanocrystals (Chen et al., 2015a; Wang et al., 2016), copper oxide (Kas et al., 2014; Ren et al., 2015; Mistry et al., 2016), copper electrodes (Ba et al., 2014; Xiao et al., 2016), and copper nanoparticles (Raciti et al., 2015; Kwon et al., 2016; Loiudice et al., 2016; Song et al., 2016). The Guido Mul group reported that the higher selectivity of ethylene for Cu nanoparticles was due to 1) a high local current density, 2) a low electrolyte concentration (i.e., buffer capacity), and 3) a high CO_2_ pressure. The sizes of nanoparticles affect their catalytic activity (Kas et al., 2015; Kas et al., 2016). The Bao group reported an electrochemical reduction of CO_2_ using different sizes of palladium nanoparticles in the range of 2.4–10.3 nm. The DFT studies have concluded that the edges and the corner sites of Pd nanoparticles were more effective in the reduction of CO_2_ than the terrace site. However, the competitive HER rate was similar for all three sites (Gao et al., 2015).

Numerous reports were made on electrochemical CO_2_ reduction using nanoparticles (Ding et al., 2016; Kim et al., 2016). Zn dendrites were used as electrocatalysts for CO conversion from CO_2_ by the Jiao group (Rosen et al., 2015a). Their catalytic activity in the aqueous bicarbonate electrolyte was threefold higher than the bulk Zn counterparts. The crystal structure of Zn was manipulated over the years to control product selectivity (Won et al., 2016). Sn dendrite electrodes were also reported as effective for the reduction of CO_2_ to formate (Won et al., 2015). The activity of the heat-treated electrode was superior and produced formate (228.6 μmol·h^−1^ cm^−2^ at −1.36 V). The reduced electrode SnO_x_/Sn with abundant O content could withstand highly reductive conditions and stabilize the ${CO}_{2}^{.-}$ intermediate. One of the noteworthy works on CO_2_ reduction to methanol was reported by the Norskov group. It was catalyzed by Ni-Ga intermetallic compound, Ni_5_Ga_3_ (Studt et al., 2014). The unique property of the catalyst involved the reduction of CO_2_ to methanol without producing a considerable amount of CO through the rWGS reaction (i.e., reverse Water Gas Shift reaction). The gallium-rich sites favored methanol synthesis, whereas the nickel sites participated in methylation and rWGS till the sites became self-poisoned due to CO and carbon. The higher activity of Ni_5_Ga_3_ at higher temperatures ensured a low rWGS rate. Thus, the amount of water in the gas was smaller, shifting the equilibrium of methanol up. The Koper group reported Pd-Pt bimetallic nanoparticles for the formation of formic acid in CO_2_. These had an onset potential of 0 V *vs*. RHE. They displayed an FE of 88% at a current density of 5 mA/cm^2^ after 1 h of electrolysis at a potential of −0.4 V *vs*. RHE (Kortlever et al., 2015). The Koper group reported Pd–Au electrocatalyst to tune the binding energy of CO as CO bonded strongly with Pd and weakly with Au (Kortlever et al., 2016). The catalyst generated a mixture of C1–C5 hydrocarbon products at an onset potential of −0.8 V *vs*. RHE due to the polymerization of the −CH_2_ group adsorbed on the catalyst surface. The Takanabe group reported Cu–In alloy for electrochemical CO_2_ reduction. The catalyst suppressed the reduction of H^+^ and promoted the reduction of aqueous CO_2_. The DFT studies for the catalyst hd revealed that In occupied the edges, and although the d-electrons of Cu remained intact, the adsorption properties were perturbed due to In (Rasul et al., 2015). Several reports were also made on using alloys for electrochemical CO_2_ reduction (Aljabour et al., 2016; Choi et al., 2016b; Larrazábal et al., 2016; Torelli et al., 2016).

The Bouwan group reported electrocatalytic CO_2_ conversion to oxalate using a tetranuclear copper (II) complex (Angamuthu et al., 2010). Several works also reported effective CO evolution activity using bismuth-based catalysts (Ding et al., 2016; Sun et al., 2016; Zhang et al., 2016). The Wang group reported a molecular Cu–porphyrin complex (copper(II)-5,10,15,20-tetrakis-(2,6-dihydroxyphenyl)porphyrin) as an electrocatalyst for CO_2_ reduction to hydrocarbons (Weng et al., 2016). At the potential of −0.976 V *vs*. RHE, the catalyst produced methane and ethylene at the partial current densities of 13.2 and 8.4 mA·cm^−2^. Another notable work in this regard was reported by the Dyer group, in which 6,7-dimethyl-4-hydroxy-2-mercaptopteridine were used to catalyze the reduction of CO_2_ on a glassy carbon electrode (Xiang et al., 2014). The bulk electrolysis of the saturated CO_2_ solution using RVC as a working electrode produced methanol in the presence of PTE. A transient intermediate, PTE carbamate was characterized by FTIR, followed by 2e^−^ reduction of CO_2_ to HCOOH, HCHO, and CH_3_OH. The Clifford P. Kubiak group (Machan et al., 2014b) reported electrocatalytic CO_2_ reduction using Re(I) bipyridine complex in which the methyl acetamidomethyl groups were added to the 4,4′-position of a 2,2′-bipyridyl ligand (Machan et al., 2014a). The complex catalyzed the reductive disproportionation of CO_2_ to CO at a lower potential, as low as 250 mV. The TOF and faradaic efficiency was low for the process. However, the reported work was a rare example of the use of hydrogen bonding and several other driving forces for molecular assembly of abiotic catalysis. [Co^III^N_4_H(Br)_2_]^+^ (N_4_H = 2,12-dimethyl-3,7,11,17-tetraazabicyclo-[11.3.1]-heptadeca-1(7),2,11, 13,15-pentaene) was reported as a catalyst in acetonitrile under glassy carbon working electrode for electrocatalytic CO_2_ reduction. The CO was generated as a product with a faradaic efficiency of 45% ± 6.4 near Co^1/0^ redox couple for [Co^III^N_4_H(Br)_2_]^+^ (E_1/2_ = −1.88 V FeC_p2_^+/0^) with concomitant hydrogen evolution with a faradaic efficiency of 30% ± 7.8 while using water as a proton source. In fact, the DFT studies concluded that Co^II^ ions were antiferromagnetically coupled to N_4_H^**.−**^ and N_4_^. **−**^, and N_4_H^**. −**^ radical anion was stable, and its ability to accommodate a second electron would result in the reduction of CO_2_. The Marinescu group reported cobalt contained a macrocycle based on azacalix [4](2,6) pyridines for reducing CO_2_ to CO (Chapovetsky et al., 2016). The presence of the NH- group led to a positive shift in the reduction potential of the Co^+1/0^ couple, which indirectly decreased the overpotential for CO_2_ reduction. Numerous studies were based on cobalt-mediated electrochemical CO_2_ reduction (Lacy et al., 2014; Morlanés et al., 2016).

The Bocarsly group (Detweiler et al., 2014) suggested MnBr(6-(2-hydroxyphenol)-2,2′-bipyridine) (CO)_3_ as an effective catalyst for the reduction of CO_2_ to CO, which bore a ligand structure with a phenolic proton in close proximity to the CO_2_ site, which allowed proton assisted C-O bond cleavage. The reduction occurred at an overpotential as low as 440 mV (Agarwal et al., 2015). Molecular manganese complexes, both immobilized and non-immobilized, have been a popular choice over the years. Ma et al. (2016a) presented a popular cAn interesting work that explored the electrochemical activity of pyridinium ion (pyrH^+^) and pyridine (py) on gold electrodes in both nitrogen and CO_2_ environment. The electrochemistry of pyridinium reduction depended on pH. The addition of CO_2_ saw an increased current density at the same potential displayed for pyridinium reduction (Lucio and Shaw, 2015). The Haywood group reported pyridine-catalyzed CO_2_ reduction to methanol. The bulk electrolysis in the galvanostatic and potentiostatic regimes occurred under pressure of 55-bar CO_2_, resulting in the formation of methanol with an FE of 10% as the charge of 5–10 C·cm^−2^ was passed (Haywood et al., 2016). The group 6 complexes of [M(CO)_4_ (bpy)] were also employed as efficient electrocatalysts by the Frantisek Hartl group (Tory et al., 2015). They could be used as electrocatalysts for the reduction of CO_2_ because the reduction potential of CO_2_ was less negative than that of radical anion [M(CO)_4_ (bpy)]^**.−**^. The cyclic voltammetry studies revealed that five-coordinate [M(CO)_3_ (bpy)]^2**−**^ was the active catalyst in the presence of Au as a working electrode. Similarly, there were also reports on tetracarbonyl complexes of low valent group 6 transition metals with diimine bidentate ligands, M(CO)_4_ (diamine), working as an efficient homogenous catalyst for electrochemical reduction of CO_2_ in non-aqueous media. The Walensky group reported a similar (α-diimine)M(CO)_3_Br (M = Mn, Re) complex for the electrochemical conversion of CO_2_ to CO (Vollmer et al., 2015). Notable work was reported by the Carter group on the influence of weak Bronsted acid (H_2_O, phenol, MeOH, and TFE) on homogenous electrocatalysts, such as [Re (bpy) (CO)_3_]^−^ and [Mn (bpy) (CO)_3_] ^−^. The TOF varied for different Brønsted acids. Moreover, for the same Brønsted acids, the TOF was higher for Re catalysts than for Mn catalysts. The order of Brønsted acid based on the TOF was TFE > MeOH > H_2_O (Riplinger and Carter, 2015). The Surendranath group (Hall et al., 2015) reported the synthesis of active graphitic surface for catalysis through condensation of *fac*-Re(5,6-diamino-1,10-phenanthroline)(CO)_3_Cl complexes with surface o-quinone moieties as an electrocatalyst for CO_2_ reduction to CO. The catalyst surface (GCC-Re surfaces) consisted of a uniform arrangement of Re centers wherein local coordination sites act as local coordination site (Oh et al., 2016).

Interestingly, the Grubbs and Gray group reported brush polymer ion gels comprising 1-butyl-3-methyl-imidazolium, PS-PEO-PS triblock brush polymer, bis-(trifluoromethylsulfonyl) imide (BMIM-TFSI), Re(bpy)(CO)_3_Cl, ferrocene (Fc), and cobaltocenium (Co $Cp_{2}^{*}$ ), as an electrocatalyst for CO_2_ reduction to CO with an FE of 90% in a non-aqueous solvent at a reduction potential of 450 mV positive of onset. The electrochemical measurements were performed using a silver pseudo-reference electrode inserted into the gel (McNicholas et al., 2016).

Another popular class of catalysts for electrochemical CO_2_ reduction was carbine-based complexes. The Wolf and Patrick group reported a range of lutidine- and pyridine-based bis-N-heterocyclic carbene (NHC) palladium pincer (Osadchuk et al., 2016; Stanton et al., 2016) complexes (Therrien et al., 2014; Therrien et al., 2015). The lutidine-based complex reduced CO_2_ to CO at a potential of −1.6 V *vs*. Ag/AgNO_3_ in the presence of 2,2,2-trifluoroacetic acid (TFA) as the proton source. The DFT studies have beautifully predicted the requirement of the proton source in the electrochemical system with respect to the degree of activation of CO_2_ and threw some light on the charge transfer dynamics of the electrocatalyst with respect to CO_2._

The Hupp, Farha, and Kubiak group reported Fe_MOF-525, a metal-organic framework for the electrochemical reduction of CO_2_ to CO (Hod et al., 2015). The MOF was reportedly stable during the CV experiments, and the CO_2_-saturated solution exhibited a catalytic wave at and before the potential of the catalytically active Fe(I/0) couple. The controlled potential electrolysis (CPE) at an overpotential of ∼650 mV yielded a current density of a few to several milliamperes. Ag/AgCl/KCl (saturated) was used as a reference electrode. A highly selective electrochemical reduction of CO_2_ to CO was reported at a pH of 4.3 with an overpotential of 480 mV using a pyrene appended iron triphenyl porphyrin ring carrying six pendant OH groups on the phenyl ring in all ortho and ortho’ positions immobilized on carbon nanotubes (Lu et al., 2016) through non-covalent interactions (Maurin and Robert, 2016). Thus, it could be deduced that Fe-porphyrin was a quite popular catalyst for electrochemical CO_2_ reduction (Choi et al., 2016a; Wu et al., 2016a). Co-based MOF, Al_2_(OH)_2_TCPP-Co, was reported as an electrocatalyst for CO_2_ reduction to CO by the Yaghi group. The MOF-integrated catalytic system (Kang et al., 2016) generated greater turnover with stability for a greater duration. The spectrochemical studies showed the reduction of Co (II) to Co (I) during catalysis (Kornienko et al., 2015). The Jean-Michel Savéant group developed an excellent catalyst for the electrochemical conversion of CO_2_ to CO using electrogenerated Fe^0^ porphyrin synthesized by substituting two opposite phenyl rings in tetraphenylporphyrin with ortho, ortho’- phenol groups, whereas the other two were perfluorinated (Costentin et al., 2014). The Ott group developed a protocol for CO formation using a methyl group in the ruthenium–bipyridine catalyst [Ru(tBu_3_tpy)(bpy)(NCCH_3_)]^2+^ to enable CO_2_ existing at a one-electron reduced state to enter an inaccessible cycle (Johnson et al., 2016). Other works investigated the energy of electrochemical CO_2_ reduction on metal-porphyrin/bipyridine motifs (Cheng et al., 2015; Ding et al., 2016; Larrazábal et al., 2016; Shen et al., 2016).

One of the notable works on electrochemical CO_2_ reduction was conducted by the Yasuaki Einaga group, in which they reduced CO_2_ in seawater using boron doped-diamond electrodes (BDD) (Nakata et al., 2014). The method was advantageous because it overcame the problem of low yield for higher-order products and reduced the generation of H_2_. The BDD electrons were used owing to their high faradaic efficiency for the production of formaldehyde (around 74%). The high faradaic efficiency is due to the sp^3^-bonded carbon of BDD. There were also reports on CO_2_ reduction in EMIM–BF_4_ ionic liquid medium at BDD modified by Cu nanoparticles (Roy et al., 2016). The size of Cu nanoparticles deposited at the BDD electrodes was 30 nm. The Hongtao Yu group reported a non-metallic catalyst and an N-doped nanodiamond/Si rod array (NDD/Si RA) for CO_2_ reduction. The catalyst led to the formation of formate from CO_2_ at an onset potential of −0.36 V (*vs*. RHE). The FE was approximately 91% at −0.8 V to −1.0 V (Liu et al., 2015). The Purkait group reported electrochemical carbon dioxide reduction coupled with the removal of dye (oxidation) using a Co_3_O_4_ electrode (Gao et al., 2016) (anode) and Sn and Zn electrocatalysts (cathode) (Yadav and Purkait, 2015). The reaction was performed in the presence of NaHCO_3_ and KHCO_3_ as electrolytes. The cyclic voltammetry data were collected over a range of applied potentials (2–3.8 V). This applied potential helped not only in the formation of HCOOH but also in the removal of the dye.

Graphenes have immense potential as electrocatalysts for electrochemical CO_2_ reduction (Wu et al., 2016a). The Hashimoto and Kamiya group reported Ni-N-modified graphene in which the Ni-N structure exhibited high catalytic activity toward CO_2_ reduction to CO (Su et al., 2016). In fact, the competitive HER rate for Ni-N-Gr was less compared to the nickel electrode. The EXAFS and XPS revealed that the introduction of Ni atoms coordinated to N into the sp^2^ network of graphene needed heat treatment and Ni-N was the active center. A high selectivity toward ethanol during CO_2_ reduction was obtained for Cu nanoparticle/N-doped graphene with an FE of 63% and selectivity of 84%. Bimetallic nanoparticles (Sarfraz et al., 2016), Pd-Cu, were dispersed on the graphene surface to catalyze the reduction of CO_2_ (Liu et al., 2016)_._ The samples with 1 wt% Pd–2wt% Cu/graphene displayed the lowest overpotential and higher current density. Clearly, graphene or catalyst-supported graphenes were a popular choice for electrochemical carbon dioxide reduction (Poh et al., 2014). The Peter Strasser group metal-doped the N-carbon material as an efficient catalyst for the conversion of CO_2_ to CO. The metal-doped N-carbon material was observed to exceed the mass activity of the carbon-supported Au catalyst by 80% at −0.5 V_RHE_ (Varela et al., 2015)_._ Along similar lines, the Xiao Dong Zhou group (Wu et al., 2014) reported an article on understanding the defect, defect density, and selectivity for N-doped carbon nanotubes in the field of electrochemical CO_2_ reduction (Sharma et al., 2015). In fact, the presence of pyridinic- and graphitic-nitrogen led to a significant decrease in overpotential (−0.18 V) and an increase in selectivity toward CO. The Lou and Ajayan group synthesized heavily nitrogen-doped carbon nanotubes using the CVD method that ensured total N-content to be 5 atom%. The maximum FE for NCNT was calculated as 80% at an overpotential of −0.26 V (Wu et al., 2015). Solvents and ionic liquids also modulated the course of electrochemical CO_2_ reduction (Grills et al., 2014; Oh et al., 2014; Sun et al., 2014; Gurkan et al., 2015; Chen et al., 2016a).

Recently, scientists discovered that to solve the CO_2_ emission problem and attain sustainability, one must convert it back to fuels. Now, this process requires energy and a good catalyst. The search for good catalysts resulted in metallic copper being a suitable system that can accomplish this job. However, there are certain limitations: they lack selectivity, H_2_ evolution is a trivial competitor, and sometimes they are operational at a higher overpotential (Hahn et al., 2017). Moreover, modeling the 2D/3D Cu/Cu_2_O surfaces is computationally costly, causing serious scientific concerns regarding the development of the catalysts (Garza et al., 2018). Here, the family of molecular metal complexes came into play with a facile synthetic methodology, control over the catalyst design, spectroscopic signatures, and ease of computational modeling (Hiroto et al., 2017). Thus, we focused our research attention on a well-known class of catalysts: corroles.

Molecular catalysts have been in development for a long time, but they have suffered from two major drawbacks: 1) C-C step-up chemistry fails (Cope et al., 2017) and 2) difficult catalyst recyclability and product recovery (Costentin et al., 2015; Rao et al., 2017). Thus, we focus on nanoscale heterogenized molecular systems such as cobalt phthalocyanine immobilized over multi-walled carbon nanotubes where remarkable activity for CO_2_ reduction to CO was observed at an overpotential of 0.52 V with a FE_CO_ >95%, and the nanoscale heterogenization effect resulted in obtaining a high current density of 15 mAcm^−2^ in the neutral aqueous medium. All the potentials were measured against Ag/AgCl reference electrode (Zhang et al., 2017). Similarly, the M. T. M. Koper group studied the immobilization of Co protoporphyrin on a pyrolytic graphite electrode. With CO_2_ electroreduction, they not only achieved volumes of CO but also minor products such as 6e^−^ reduced methanol and 8e^−^ reduced methane at a relatively low overpotential of 0.5 V. The potential was measured using RHE as the reference electrode. Their work also provided insights into controlling the selectivity of CO_2_ reduction by suppressing H_2_ evolution and the pH effect in controlling CO formation over H_2_ evolution, indicating the formation of Brønsted base type intermediate (carboxyhydroxyl intermediate) (Shen et al., 2015). The Hailiang Wang group explored Cu(II) porphyrin immobilized over the carbon paper electrode, which showed increased CO_2_ reducibility to hydrocarbons, such as methane and ethylene at −0.976 V *vs*. RHE (Weng et al., 2016). Corroles with structural similarity to porphyrins were a suitable candidate for electrochemical CO_2_ reduction, which was first explored by the Zeev Gross and E. Fujita group. Co and Fe corroles were investigated under homogeneous conditions, resulting in CO formation. Their studies revealed that the Co(I) and Fe(I) states of the catalysts formed under electrochemical bias are responsible for CO_2_ reduction (Grodkowski et al., 2002).

**FIGURE 9:**
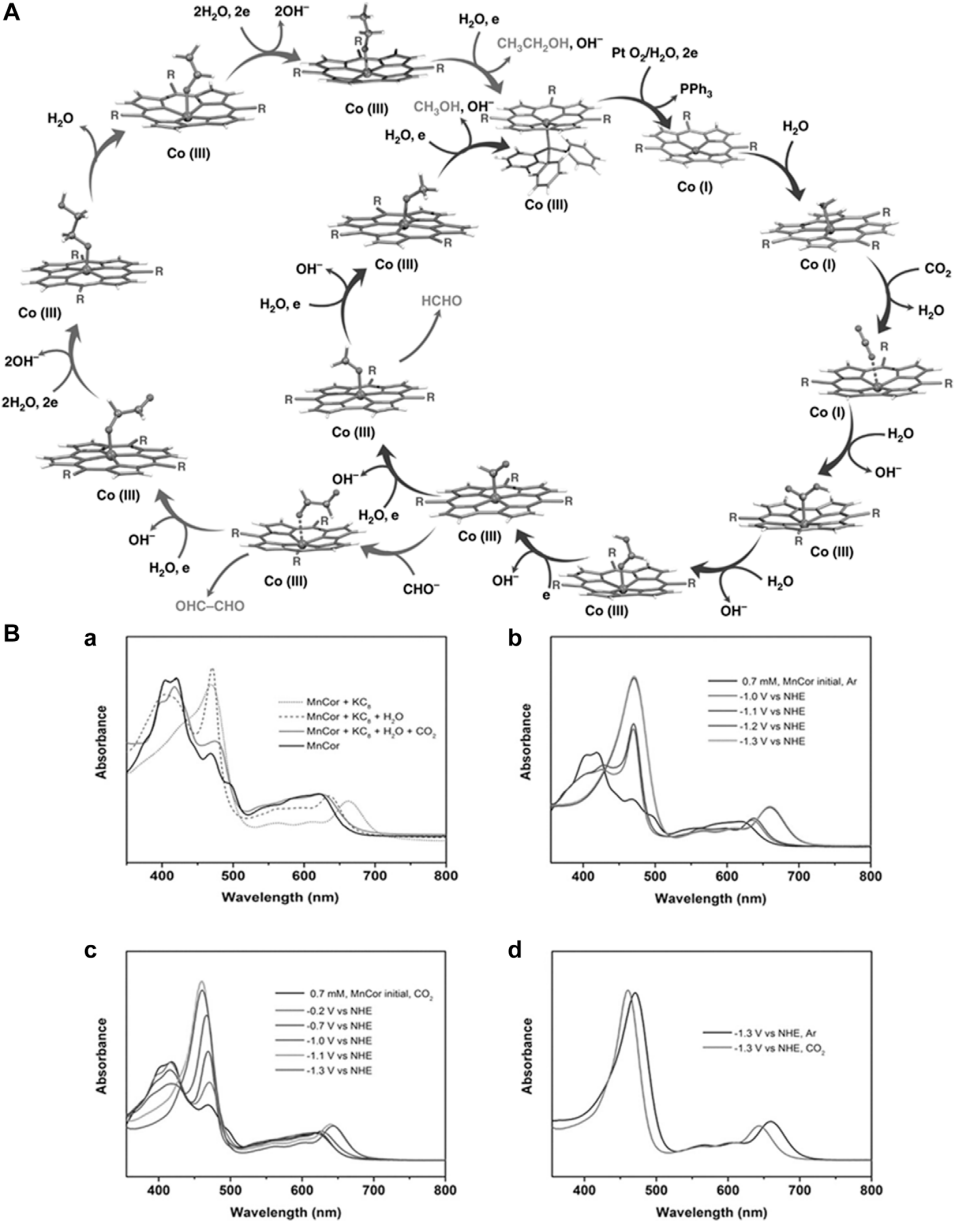
**(A)** Proposed single site mechanism of CO_2_ reduction using Co corrole. **(B)** (a) EAS of Mn corrole in acetonitrile under argon (black solid line), the chemically reduced form in the presence of KC_8_ (red dotted line), after the addition of water (blue, dashed line), and subsequent dosage of CO_2_ (green solid line). (b–d) Potential dependent SEC-UV/Vis of 0.7 mm Mn corrole in acetonitrile with 2% water and 0.2 M TBAPF_6_ as electrolyte after 2 min CPE (b) under argon, (c) under CO_2_, and (d) comparison of UV/Vis spectra observed at −1.3 V *vs*. NHE under argon (black) and CO_2_ dosage (red). SEC-UV/V measurements were recorded with a light transparent platinum mini-grid as working, as counter, and an Ag-microwire as a pseudo-reference electrode.

**FIGURE 10:**
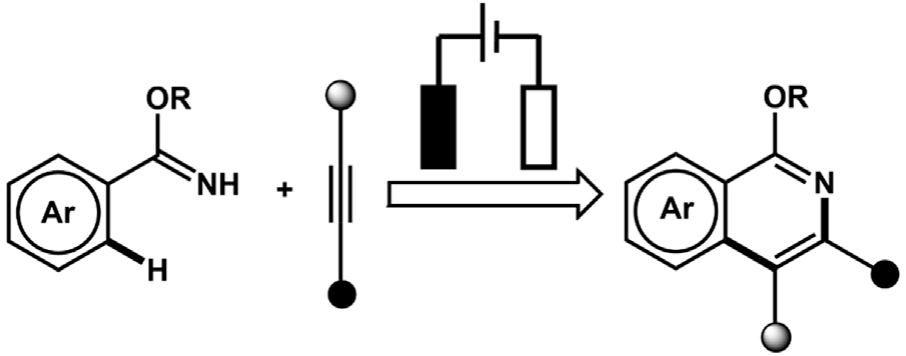
A representative diagram for electro-catalyzed C-H annulations.

Our collaboration and the synthetic excellence of Professor Wolfgang Schӧfberger resulted in a (–S-PEG(7)-OMe)_3_ modified Co(III)-corrole, which led to a 12e reduction of CO_2_ to ethanol electrochemically at a low potential of −0.8 V *vs*. RHE (Gonglach et al., 2019). Systematic spectroscopic investigation and DFT calculation led to the proposition that shuttling back and forth between Co(III) and Co(I) states under electrochemical bias follows two divergent reaction pathways: one leading to the 6e reduced product methanol and the other 12e reduced product ethanol as shown in Figure 9. The low operational potential of the catalysts and formation of highly reduced products with C-C step-up was due to the formation of easily reducible glyoxal type intermediate (as shown in Figure 10). Now, to understand the mode of action of the metal center in the catalysis, Mn(III)-corrole was designed with the help of (–S-PEG(7)-OMe)_3_ modification (De et al., 2020). The previously supported Mn metallocomplexes showed excellent CO_2_ reducibility as in the case of Mn(bipyridine)-pyrene complexes where [MnBr(2,2′-bipyridine)(CO)_3_] was anchored over carbon nanotubes using pyrene moieties. High surface loading of the Mn complex led to the formation of Mn(0) dimer under electrochemical bias, reducing CO_2_ to CO. However, for low surface loading, Mn hydride formation shifted the reaction to a formic acid pathway (Reuillard et al., 2017). The A. J. Cowan group studied the same with different Mn-bipyridine carbonyl complexes immobilized over multi-walled carbon nanotubes (MWCNT) (Walsh et al., 2014; Walsh et al., 2015). The S. Sato group studied the Mn complex immobilized MWCNT and established a relationship between heterogeneous electrochemical CO_2_ reduction and the promotional effect of K^+^ ions. They found excellent stability of the electrodes under electrochemical conditions for at least 48 h with a current density of 2.0 mAcm^−2^ at −0.39 V *vs*. RHE. The significant lowering of the overpotential was a result of the electron storage properties of the MWCNTs and surface adsorption of K^+^, promoting the adsorption of CO_2_ over the catalysts (Sato et al., 2018). Based on the above experimental and theoretical support, our group conducted CO_2_ electroreduction over Mn(III)-corrole (–S-PEG (7)-OMe)_3_ immobilized over carbon paper electrodes. The 8e reduction of CO_2_ to acetate resulted from a faradaic efficiency of 63% and TOF of 8.25 h^−1^ (De et al., 2020). Comparing both the CO_2_ reduction results (Co(III) and Mn(III)-corrole) and tallying the chemical reduction study with density functional theory calculations, we reached the conclusion that the catalysis was metal-centered with the corrole ligand stabilizing multiple redox intermediates.

## 5 Future perspective

The use of electricity to achieve chemical transformations, which traditionally involve cumbersome steps, including a stoichiometric amount of reagents, has opened up a whole new avenue in the field of catalysis. The field of electrosynthesis is evolving with the adoption of new concepts and strategies from other fields, such as magneto-electrochemistry (Dong et al., 2014; Vock et al., 2015; Clausmeyer et al., 2016) and ultrasonication (Atobe, 2014). Innovation in electrolyte and electrode systems is needed constantly to circumvent the limitation of the current electrode materials, such as mercury lead. The modern electrode system should be resistant to corrosion and should provide a large overpotential for undesired side reactions. BDE electrodes and synthetic carbon allotropes with modified surfaces have proven to be a step in a similar direction. There should also be enough emphasis on investigating solvents not only as a reaction medium but as a tool to moderate selectivity in the electrosynthetic pathway. There is also a vast scope for development in other aspects of electrochemical synthesis such as supporting electrolytes and electrolysis cells. The sustainability of electrochemical reactions might popularize it as a common synthetic method rather than a niche technology.

## 6 Conclusion

In this review, we have discussed different factors driving electrochemical reactions and have summarized different endeavors of electrochemical organic reaction and electrochemical CO_2_ reduction by several groups across the world. In order to perform a successful electrochemical reaction, the major challenges have been the generation and trapping of radical cations, current optimization, and the development of site-selective reactions on a microelectrode array. Over the years, we have been able to oxidize and reduce an array of substrates through these electrochemical reactions. This approach has been utilized over the years to drive reactions in a simple photovoltaic cell to a simple battery to preparative electrolysis setups to a microelectrode array. It is difficult for any other approach to explore such a varied scope of chemistry, which has been explored by electrochemical studies.

## References

[B1] Adams R. N. (1969). Anodic oxidation pathways of aromatic hydrocarbons and amines. Acc. Chem. Res. 2, 175–180. 10.1021/ar50018a003

[B2] Adeli Y. Huang K. Liang Y. Jiang Y. Liu J. Song S. (2019). Electrochemically oxidative C–C bond cleavage of alkylarenes for anilines synthesis. ACS Catal. 9, 2063–2067. 10.1021/acscatal.8b04351

[B3] Agarwal J. Shaw T. W. Schaefer H. F. Iii Bocarsly A. B. (2015). Design of a catalytic active site for electrochemical CO2 reduction with Mn (I)-tricarbonyl species. Inorg. Chem. 54, 5285–5294. 10.1021/acs.inorgchem.5b00233 25968283

[B4] Aljabour A. Apaydin D. H. Coskun H. Ozel F. Ersoz M. Stadler P. (2016). Improvement of catalytic activity by nanofibrous CuInS2 for electrochemical CO2 reduction. ACS Appl. Mat. Interfaces 8, 31695–31701. 10.1021/acsami.6b11151 27802019

[B5] Allen B. D. Hareram M. D. Seastram A. C. Mcbride T. Wirth T. Browne D. L. (2019). Manganese-catalyzed electrochemical deconstructive chlorination of cycloalkanols via alkoxy radicals. Org. Lett. 21, 9241–9246. 10.1021/acs.orglett.9b03652 31687826 PMC7007279

[B182] Angamuthu R. Byers P. Lutz M. Spek A. L. Bouwman E. (2010). Electrocatalytic CO_2_ conversion to oxalate by a copper complex. Science 327 (5963), 313–315. 20075248 10.1126/science.1177981

[B6] Asadi M. Kumar B. Behranginia A. Rosen B. A. Baskin A. Repnin N. (2014). Robust carbon dioxide reduction on molybdenum disulphide edges. Nat. Commun. 5, 4470–4478. 10.1038/ncomms5470 25073814

[B7] Atobe M. (2014). “Electrosynthesis under ultrasound and centrifugal fields,” in Encyclopedia of applied electrochemistry (Berlin, Germany: Springer).

[B8] Ba X. Yan L.-L. Huang S. Yu J. Xia X.-J. Yu Y. (2014). New way for CO2 reduction under visible light by a combination of a Cu electrode and semiconductor thin film: Cu2O conduction type and morphology effect. J. Phys. Chem. C 118, 24467–24478. 10.1021/jp5063397

[B9] Badalyan A. Stahl S. S. (2016). Cooperative electrocatalytic alcohol oxidation with electron-proton-transfer mediators. Nature 535, 406–410. 10.1038/nature18008 27350245

[B10] Barker G. Gardner A. (1960). Pulse polarography. Z. Anal. Chem. 173, 79–83. 10.1007/bf00448718

[B11] Barker G. Gonsalves M. Macpherson J. Slevin C. Unwin P. (1958). Square wave polarography and some related techniques. Anal. Chim. Acta 18, 118–131. 10.1016/s0003-2670(00)87111-1

[B12] Baturina O. A. Lu Q. Padilla M. A. Xin L. Li W. Serov A. (2014). CO2 electroreduction to hydrocarbons on carbon-supported Cu nanoparticles. ACS Catal. 4, 3682–3695. 10.1021/cs500537y

[B13] Beil S. B. Müller T. Sillart S. B. Franzmann P. Bomm A. Holtkamp M. (2018). Active molybdenum‐based anode for dehydrogenative coupling reactions. Angew. Chem. Int. Ed. 57, 2450–2454. 10.1002/anie.201712718 29318724

[B14] Bergens S. H. Gorman C. B. Palmore G. T. R. Whitesides G. M. (1994). A redox fuel cell that operates with methane as fuel at 120 C. Science 265, 1418–1420. 10.1126/science.265.5177.1418 17833814

[B15] Bockris J. O. M. Conway B. E. White R. E. (1992). Modern aspects of electrochemistry. Berlin, Germany: Springer Science & Business Media.

[B16] Borman S. (1982). New electroanalytical pulse techniques. Anal. Chem. 54, A698.

[B17] Bryan M. C. Dillon B. Hamann L. G. Hughes G. J. Kopach M. E. Peterson E. A. (2013). Sustainable practices in medicinal chemistry: Current state and future directions. J. Med. Chem. 56, 6007–6021. 10.1021/jm400250p 23586692

[B18] Cai C.-Y. Xu H.-C. (2018). Dehydrogenative reagent-free annulation of alkenes with diols for the synthesis of saturated O-heterocycles. Nat. Commun. 9, 3551–3557. 10.1038/s41467-018-06020-8 30177691 PMC6120897

[B19] Cardiel A. C. Taitt B. J. Choi K.-S. (2019). Stabilities, regeneration pathways, and electrocatalytic properties of nitroxyl radicals for the electrochemical oxidation of 5-hydroxymethylfurfural. ACS Sustain. Chem. Eng. 7, 11138–11149. 10.1021/acssuschemeng.9b00203

[B20] Cernak T. Dykstra K. D. Tyagarajan S. Vachal P. Krska S. W. (2016). The medicinal chemist's toolbox for late stage functionalization of drug-like molecules. Chem. Soc. Rev. 45, 546–576. 10.1039/c5cs00628g 26507237

[B21] Chai G.-L. Guo Z.-X. (2016). Highly effective sites and selectivity of nitrogen-doped graphene/CNT catalysts for CO 2 electrochemical reduction. Chem. Sci. 7, 1268–1275. 10.1039/c5sc03695j 29910883 PMC5975832

[B22] Chan K. Tsai C. Hansen H. A. Nørskov J. K. (2014). Molybdenum sulfides and selenides as possible electrocatalysts for CO2 reduction. ChemCatChem 6, 1899–1905. 10.1002/cctc.201402128

[B23] Chapovetsky A. Do T. H. Haiges R. Takase M. K. Marinescu S. C. (2016). Proton-assisted reduction of CO2 by cobalt aminopyridine macrocycles. J. Am. Chem. Soc. 138, 5765–5768. 10.1021/jacs.6b01980 27092968

[B24] Chen C. S. Handoko A. D. Wan J. H. Ma L. Ren D. Yeo B. S. (2015a). Stable and selective electrochemical reduction of carbon dioxide to ethylene on copper mesocrystals. Catal. Sci. Technol. 5, 161–168. 10.1039/c4cy00906a

[B25] Chen J. Lv S. Tian S. (2019). Electrochemical transition‐metal‐catalyzed C− H bond functionalization: Electricity as clean surrogates of chemical oxidants. ChemSusChem 12, 115–132. 10.1002/cssc.201801946 30280508

[B26] Chen J. Yan W.-Q. Lam C. M. Zeng C.-C. Hu L.-M. Little R. D. (2015b). Electrocatalytic aziridination of alkenes mediated by n-bu4NI: A radical pathway. Org. Lett. 17, 986–989. 10.1021/acs.orglett.5b00083 25654310

[B27] Chen L. D. Urushihara M. Chan K. Nørskov J. K. (2016b). Electric field effects in electrochemical CO2 reduction. ACS Catal. 6, 7133–7139. 10.1021/acscatal.6b02299

[B28] Chen L. Guo S. X. Li F. Bentley C. Horne M. Bond A. M. (2016a). Electrochemical reduction of CO2 at metal electrodes in a distillable ionic liquid. ChemSusChem 9, 1271–1278. 10.1002/cssc.201600359 27164263

[B29] Cheng M.-J. Kwon Y. Head-Gordon M. Bell A. T. (2015). Tailoring metal-porphyrin-like active sites on graphene to improve the efficiency and selectivity of electrochemical CO2 reduction. J. Phys. Chem. C 119, 21345–21352. 10.1021/acs.jpcc.5b05518

[B30] Cheng T. Xiao H. Goddard W. A. Iii (2016). Reaction mechanisms for the electrochemical reduction of CO2 to CO and formate on the Cu (100) surface at 298 K from quantum mechanics free energy calculations with explicit water. J. Am. Chem. Soc. 138, 13802–13805. 10.1021/jacs.6b08534 27726392

[B31] Chiba K. Kim S. (2009). Anodic carbon-carbon bond formation in lithium perchlorate/nitromethane electrolyte solution. Electrochemistry 77, 21–29. 10.5796/electrochemistry.77.21

[B32] Choi J. Benedetti T. M. Jalili R. Walker A. Wallace G. G. Officer D. L. (2016a). High performance Fe porphyrin/ionic liquid Co‐catalyst for electrochemical CO2 reduction. Chem. Eur. J. 22, 14158–14161. 10.1002/chem.201603359 27464300

[B33] Choi S. Y. Jeong S. K. Kim H. J. Baek I.-H. Park K. T. (2016b). Electrochemical reduction of carbon dioxide to formate on tin–lead alloys. ACS Sustain. Chem. Eng. 4, 1311–1318. 10.1021/acssuschemeng.5b01336

[B34] Claraz A. L. Courant T. Masson G. R. (2020). Electrochemical intramolecular oxytrifluoromethylation of N-tethered alkenyl alcohols: Synthesis of functionalized morpholines. Org. Lett. 22, 1580–1584. 10.1021/acs.orglett.0c00176 32017576

[B35] Clausmeyer J. Wilde P. Löffler T. Ventosa E. Tschulik K. Schuhmann W. (2016). Detection of individual nanoparticle impacts using etched carbon nanoelectrodes. Electrochem. Commun. 73, 67–70. 10.1016/j.elecom.2016.11.003

[B36] Conway B. E. Bockris J. M. (2012). Modern aspects of electrochemistry: No. 9. Berlin, Germany: Springer Science & Business Media.

[B37] Cope J. D. Liyanage N. P. Kelley P. J. Denny J. A. Valente E. J. Webster C. E. (2017). Electrocatalytic reduction of CO 2 with CCC-NHC pincer nickel complexes. Chem. Commun. 53, 9442–9445. 10.1039/c6cc06537f 28792548

[B38] Costentin C. Passard G. Robert M. Savéant J.-M. (2014). Ultraefficient homogeneous catalyst for the CO2-to-CO electrochemical conversion. Proc. Natl. Acad. Sci. U. S. A. 111, 14990–14994. 10.1073/pnas.1416697111 25288744 PMC4210317

[B39] Costentin C. Robert M. SavéAnt J.-M. (2015). Current issues in molecular catalysis illustrated by iron porphyrins as catalysts of the CO2-to-CO electrochemical conversion. Acc. Chem. Res. 48, 2996–3006. 10.1021/acs.accounts.5b00262 26559053

[B40] Das A. Nutting J. E. Stahl S. S. (2019). Electrochemical C–H oxygenation and alcohol dehydrogenation involving Fe-oxo species using water as the oxygen source. Chem. Sci. 10, 7542–7548. 10.1039/c9sc02609f 31588305 PMC6761876

[B41] Das A. Stahl S. S. (2017). Noncovalent immobilization of molecular electrocatalysts for chemical synthesis: Efficient electrochemical alcohol oxidation with a pyrene–TEMPO conjugate. Angew. Chem. Int. Ed. Engl. 56, 9018–9023. 10.1002/ange.201704921 28586133 PMC5831151

[B42] Daugulis O. Roane J. Tran L. D. (2015). Bidentate, monoanionic auxiliary-directed functionalization of carbon–hydrogen bonds. Acc. Chem. Res. 48, 1053–1064. 10.1021/ar5004626 25756616 PMC4406856

[B43] De R. Gonglach S. Paul S. Haas M. Sreejith S. Gerschel P. (2020). Frontispiz: Electrocatalytic reduction of CO _2_ to acetic acid by a molecular manganese corrole complex. Angew. Chem. Int. Ed. Engl. 132, 202082662. 10.1002/ange.202082662 PMC7540269 32281187

[B44] Detweiler Z. M. White J. L. Bernasek S. L. Bocarsly A. B. (2014). Anodized indium metal electrodes for enhanced carbon dioxide reduction in aqueous electrolyte. Langmuir 30, 7593–7600. 10.1021/la501245p 24940629

[B45] Ding C. Li A. Lu S.-M. Zhang H. Li C. (2016). *In situ* electrodeposited indium nanocrystals for efficient CO2 reduction to CO with low overpotential. ACS Catal. 6, 6438–6443. 10.1021/acscatal.6b01795

[B46] Dong J. Huang Y. Jin L. Lin H. Yang H. (2014). Thermal optimization of a high-speed permanent magnet motor. IEEE Trans. Magn. 50, 749–752. 10.1109/tmag.2013.2285017

[B47] Dou G.-Y. Jiang Y.-Y. Xu K. Zeng C.-C. (2019). Electrochemical Minisci-type trifluoromethylation of electron-deficient heterocycles mediated by bromide ions. Org. Chem. Front. 6, 2392–2397. 10.1039/c9qo00552h

[B48] Du D. Lan R. Humphreys J. Sengodan S. Xie K. Wang H. (2016). Achieving both high selectivity and current density for CO2 reduction to formate on nanoporous tin foam electrocatalysts. ChemistrySelect 1, 1711–1715. 10.1002/slct.201600451

[B49] Eberson L. Nyberg K. (1973). Anodic aromatic substitution. Acc. Chem. Res. 6, 106–112. 10.1021/ar50063a004

[B50] Eberson L. Nyberg K. (1976). Synthetic uses of anodic substitution reactions. Tetrahedron 32, 2185–2206. 10.1016/0040-4020(76)85132-0

[B51] Edinger C. Waldvogel S. R. (2014). Electrochemical deoxygenation of aromatic amides and sulfoxides. Eur. J. Org. Chem. 2014, 5144–5148. 10.1002/ejoc.201402714

[B52] Elving P. J. Pullman B. (2009). Mechanisms of organic electrode reactions. Adv. Chem. Phys. 3, 1–31. 10.1002/9780470143490.ch1

[B53] Evans D. H. (2008). One-electron and two-electron transfers in electrochemistry and homogeneous solution reactions. Chem. Rev. 108, 2113–2144. 10.1021/cr068066l 18620364

[B54] Farghaly O. A. Hameed R. A. Abu-Nawwas A.-a. H. (2014). Analytical application using modern electrochemical techniques. Int. J. Electrochem. Sci. 9, 3287–3318.

[B55] Feng R. Smith J. A. Moeller K. D. (2017). Anodic cyclization reactions and the mechanistic strategies that enable optimization. Acc. Chem. Res. 50, 2346–2352. 10.1021/acs.accounts.7b00287 28858480

[B56] Francke R. Little R. D. (2014). Redox catalysis in organic electrosynthesis: Basic principles and recent developments. Chem. Soc. Rev. 43, 2492–2521. 10.1039/c3cs60464k 24500279

[B57] Frontana-Uribe B. A. Little R. D. Ibanez J. G. Palma A. Vasquez-Medrano R. (2010). Organic electrosynthesis: A promising green methodology in organic chemistry. Green Chem. 12, 2099–2119. 10.1039/c0gc00382d

[B58] Fu N. Sauer G. S. Saha A. Loo A. Lin S. (2017). Metal-catalyzed electrochemical diazidation of alkenes. Science 357, 575–579. 10.1126/science.aan6206 28798126

[B59] Gao D. Zhou H. Wang J. Miao S. Yang F. Wang G. (2015). Size-dependent electrocatalytic reduction of CO2 over Pd nanoparticles. J. Am. Chem. Soc. 137, 4288–4291. 10.1021/jacs.5b00046 25746233

[B60] Gao S. Jiao X. Sun Z. Zhang W. Sun Y. Wang C. (2016). Ultrathin Co3O4 layers realizing optimized CO2 electroreduction to formate. Angew. Chem. Int. Ed. Engl. 55, 708–712. 10.1002/ange.201509800 26783062

[B61] Garza A. J. Bell A. T. Head-Gordon M. (2018). Mechanism of CO2 reduction at copper surfaces: Pathways to C2 products. ACS Catal. 8, 1490–1499. 10.1021/acscatal.7b03477

[B62] Glasstone S. Hickling A. (1939). The hydrogen peroxide theory of electrolytic oxidation. Chem. Rev. 25, 407–441. 10.1021/cr60082a003

[B63] Gonglach S. Paul S. Haas M. Pillwein F. Sreejith S. S. Barman S. (2019). Molecular cobalt corrole complex for the heterogeneous electrocatalytic reduction of carbon dioxide. Nat. Commun. 10, 3864–3873. 10.1038/s41467-019-11868-5 31455766 PMC6711975

[B64] Gorman C. B. Bergens S. H. Whitesides G. M. (1996). Platinum-catalyzed oxidations of organic compounds by ferric sulfate: Use of a redox fuel cell to mediate complete oxidation of ethylene glycol by dioxygen at 80° C. J. Catal. 158, 92–96. 10.1006/jcat.1996.0009

[B65] Grills D. C. Matsubara Y. Kuwahara Y. Golisz S. R. Kurtz D. A. Mello B. A. (2014). Electrocatalytic CO2 reduction with a homogeneous catalyst in ionic liquid: High catalytic activity at low overpotential. J. Phys. Chem. Lett. 5, 2033–2038. 10.1021/jz500759x 26273891

[B66] Grimshaw J. (2000). Electrochemical reactions and mechanisms in organic chemistry. Amsterdam, Netherlands: Elsevier.

[B67] Grjotheim K. Krohn C. Malinovsky M. Matiasovsky K. Thonstad J. (1977). “Aluminum electrolysis,” in The chemistry of the Hall-heroult process (Dusseldorf, Germany: Aluminium- Verlag GmbH), 350.

[B68] Grodkowski J. Neta P. Fujita E. Mahammed A. Simkhovich L. Gross Z. (2002). Reduction of cobalt and iron corroles and catalyzed reduction of CO2. J. Phys. Chem. A 106, 4772–4778. 10.1021/jp013668o

[B69] Guo X.-X. Gu D.-W. Wu Z. Zhang W. (2015). Copper-catalyzed C–H functionalization reactions: Efficient synthesis of heterocycles. Chem. Rev. 115, 1622–1651. 10.1021/cr500410y 25531056

[B70] Gurkan B. Simeon F. Hatton T. A. (2015). Quinone reduction in ionic liquids for electrochemical CO2 separation. ACS Sustain. Chem. Eng. 3, 1394–1405. 10.1021/acssuschemeng.5b00116

[B71] Hahn C. Hatsukade T. Kim Y.-G. Vailionis A. Baricuatro J. H. Higgins D. C. (2017). Engineering Cu surfaces for the electrocatalytic conversion of CO2: Controlling selectivity toward oxygenates and hydrocarbons. Proc. Natl. Acad. Sci. U. S. A. 114, 5918–5923. 10.1073/pnas.1618935114 28533377 PMC5468660

[B72] Hall A. S. Yoon Y. Wuttig A. Surendranath Y. (2015). Mesostructure-induced selectivity in CO2 reduction catalysis. J. Am. Chem. Soc. 137, 14834–14837. 10.1021/jacs.5b08259 26536054

[B73] Hammes-Schiffer S. Soudackov A. V. (2008). Proton-coupled electron transfer in solution, proteins, and electrochemistry. J. Phys. Chem. B 112, 14108–14123. 10.1021/jp805876e 18842015 PMC2720037

[B74] Harnisch F. Schröder U. (2019). Tapping renewables: A new dawn for organic electrosynthesis in aqueous reaction media. ChemElectroChem 6, 4126–4133. 10.1002/celc.201900456

[B75] Hashiguchi B. G. Bischof S. M. Konnick M. M. Periana R. A. (2012). Designing catalysts for functionalization of unactivated C–H bonds based on the CH activation reaction. Acc. Chem. Res. 45, 885–898. 10.1021/ar200250r 22482496

[B76] Hayashi R. Shimizu A. Song Y. Ashikari Y. Nokami T. Yoshida J. I. (2017). Metal‐free benzylic C− H amination via electrochemically generated benzylaminosulfonium ions. Chem. Eur. J. 23, 61–64. 10.1002/chem.201604484 27790762

[B77] Haywood S. K. Rybchenko S. I. Touhami D. Wadhawan J. D. (2016). Study of pyridine-mediated electrochemical reduction of CO2 to methanol at high CO2 pressure. ChemSusChem 9, 1660–1669. 10.1002/cssc.201600267 27253886

[B78] He T.-J. Ye Z. Ke Z. Huang J.-M. (2019). Stereoselective synthesis of sulfur-containing β-enaminonitrile derivatives through electrochemical Csp 3–H bond oxidative functionalization of acetonitrile. Nat. Commun. 10, 833–839. 10.1038/s41467-019-08762-5 30783088 PMC6381189

[B79] Hertl W. Weetall H. (1985a). A photo-chemical electrical fuel cell: Part I. Alcohol fuels. Bioelectrochemistry Bioenergetics 14, 357–366. 10.1016/0302-4598(85)80008-8

[B80] Hertl W. Weetall H. (1985b). A photo-chemical electrical fuel cell: Part II. Carbohydrate fuels. Bioelectrochemistry Bioenergetics 14, 367–373. 10.1016/0302-4598(85)80009-x

[B81] Hilt G. (2020). Basic strategies and types of applications in organic electrochemistry. ChemElectroChem 7, 395–405. 10.1002/celc.201901799

[B82] Hiroto S. Miyake Y. Shinokubo H. (2017). Synthesis and functionalization of porphyrins through organometallic methodologies. Chem. Rev. 117, 2910–3043. 10.1021/acs.chemrev.6b00427 27709907

[B83] Hod I. Sampson M. D. Deria P. Kubiak C. P. Farha O. K. Hupp J. T. (2015). Fe-porphyrin-based metal–organic framework films as high-surface concentration, heterogeneous catalysts for electrochemical reduction of CO2. ACS Catal. 5, 6302–6309. 10.1021/acscatal.5b01767

[B84] Hong H. Li Y. Chen L. Li B. Zhu Z. Chen X. (2019). Electrochemical synthesis strategy for cvinyl-CF3 compounds through decarboxylative trifluoromethylation. J. Org. Chem. 84, 5980–5986. 10.1021/acs.joc.9b00766 31013077

[B85] Hoover J. M. Ryland B. L. Stahl S. S. (2013). Mechanism of copper (I)/TEMPO-catalyzed aerobic alcohol oxidation. J. Am. Chem. Soc. 135, 2357–2367. 10.1021/ja3117203 23317450 PMC3834274

[B86] Horn E. J. Rosen B. R. Chen Y. Tang J. Chen K. Eastgate M. D. (2016). Scalable and sustainable electrochemical allylic C–H oxidation. Nature 533, 77–81. 10.1038/nature17431 27096371 PMC4860034

[B87] Hu X. Zhang G. Bu F. Luo X. Yi K. Zhang H. (2018a). Photoinduced oxidative activation of electron-rich arenes: alkenylation with H 2 evolution under external oxidant-free conditions. Chem. Sci. 9, 1521–1526. 10.1039/c7sc04634k 29675195 PMC5887105

[B88] Hu X. Zhang G. Bu F. Nie L. Lei A. (2018b). Electrochemical-oxidation-induced site-selective intramolecular C (sp3)–H amination. ACS Catal. 8, 9370–9375. 10.1021/acscatal.8b02847

[B89] Hu X. Zhang G. Nie L. Kong T. Lei A. (2019). Electrochemical oxidation induced intermolecular aromatic CH imidation. Nat. Commun. 10, 5467–5476. 10.1038/s41467-019-13524-4 31784522 PMC6884519

[B90] Huan T. N. Prakash P. Simon P. Rousse G. Xu X. Artero V. (2016). CO2 reduction to CO in water: Carbon nanotube–gold nanohybrid as a selective and efficient electrocatalyst. ChemSusChem 9, 2317–2320. 10.1002/cssc.201600597 27492905

[B91] Huang H. Strater Z. M. Lambert T. H. (2020). Electrophotocatalytic C–H functionalization of ethers with high regioselectivity. J. Am. Chem. Soc. 142, 1698–1703. 10.1021/jacs.9b11472 31904939 PMC7251231

[B92] Inzelt G. Lewenstam A. Scholz F. (2013). Handbook of reference electrodes. Berlin, Germany: Springer.

[B93] Janz G. J. (2012). Nonaqueous electrolytes handbook. Amsterdam, Netherlands: Elsevier.

[B94] Jiang Y. Xu K. Zeng C. (2017). Use of electrochemistry in the synthesis of heterocyclic structures. Chem. Rev. 118, 4485–4540. 10.1021/acs.chemrev.7b00271 29039924

[B95] Johnson B. A. Maji S. Agarwala H. White T. A. Mijangos E. Ott S. (2016). Activating a low overpotential CO2 reduction mechanism by a strategic ligand modification on a ruthenium polypyridyl catalyst. Angew. Chem. Int. Ed. Engl. 55, 1857–1861. 10.1002/ange.201508490 26671836

[B96] Joseph W. (2000). Analytical electrochemistry. New York: Wiley VCH.

[B97] Kalvoda R. Kopanica M. (1989). Adsorptive stripping voltammetry in trace analysis. Pure Appl. Chem. 61, 97–112. 10.1351/pac198961010097

[B98] Kang X. Zhu Q. Sun X. Hu J. Zhang J. Liu Z. (2016). Highly efficient electrochemical reduction of CO 2 to CH 4 in an ionic liquid using a metal–organic framework cathode. Chem. Sci. 7, 266–273. 10.1039/c5sc03291a 29861981 PMC5952524

[B99] Kärkäs M. D. (2018). Electrochemical strategies for C–H functionalization and C–N bond formation. Chem. Soc. Rev. 47, 5786–5865. 10.1039/c7cs00619e 29911724

[B100] Kas R. Hummadi K. K. Kortlever R. De Wit P. Milbrat A. Luiten-Olieman M. W. (2016). Three-dimensional porous hollow fibre copper electrodes for efficient and high-rate electrochemical carbon dioxide reduction. Nat. Commun. 7, 10748–10757. 10.1038/ncomms10748 26888578 PMC4759634

[B101] Kas R. Kortlever R. Milbrat A. Koper M. T. Mul G. Baltrusaitis J. (2014). Electrochemical CO 2 reduction on Cu 2 O-derived copper nanoparticles: Controlling the catalytic selectivity of hydrocarbons. Phys. Chem. Chem. Phys. 16, 12194–12201. 10.1039/c4cp01520g 24817571

[B102] Kas R. Kortlever R. Yılmaz H. Koper M. T. Mul G. (2015). Manipulating the hydrocarbon selectivity of copper nanoparticles in CO2 electroreduction by process conditions. ChemElectroChem 2, 354–358. 10.1002/celc.201402373

[B103] Kathiravan S. Suriyanarayanan S. Nicholls I. A. (2019). Electrooxidative amination of sp2 C–H bonds: Coupling of amines with aryl amides via copper catalysis. Org. Lett. 21, 1968–1972. 10.1021/acs.orglett.9b00003 30785289

[B104] Kawamata Y. Yan M. Liu Z. Bao D.-H. Chen J. Starr J. T. (2017). Scalable, electrochemical oxidation of unactivated C–H bonds. J. Am. Chem. Soc. 139, 7448–7451. 10.1021/jacs.7b03539 28510449 PMC5465511

[B105] Kim C. Jeon H. S. Eom T. Jee M. S. Kim H. Friend C. M. (2015). Achieving selective and efficient electrocatalytic activity for CO2 reduction using immobilized silver nanoparticles. J. Am. Chem. Soc. 137, 13844–13850. 10.1021/jacs.5b06568 26447349

[B106] Kim D. Resasco J. Yu Y. Asiri A. M. Yang P. (2014). Synergistic geometric and electronic effects for electrochemical reduction of carbon dioxide using gold–copper bimetallic nanoparticles. Nat. Commun. 5, 4948–8. 10.1038/ncomms5948 25208828

[B107] Kim K.-S. Kim W. J. Lim H.-K. Lee E. K. Kim H. (2016). Tuned chemical bonding ability of Au at grain boundaries for enhanced electrochemical CO2 reduction. ACS Catal. 6, 4443–4448. 10.1021/acscatal.6b00412

[B108] Kingston C. Palkowitz M. D. Takahira Y. Vantourout J. C. Peters B. K. Kawamata Y. (2019). A survival guide for the “electro-curious”. Acc. Chem. Res. 53, 72–83. 10.1021/acs.accounts.9b00539 31823612 PMC6996934

[B109] Kirste A. Elsler B. Schnakenburg G. Waldvogel S. R. (2012). Efficient anodic and direct phenol-arene C, C cross-coupling: The benign role of water or methanol. J. Am. Chem. Soc. 134, 3571–3576. 10.1021/ja211005g 22242769

[B110] Kirste A. Hayashi S. Schnakenburg G. Malkowsky I. M. Stecker F. Fischer A. (2011a). Highly selective electrosynthesis of biphenols on graphite electrodes in fluorinated media. Chem. Eur. J. 17, 14164–14169. 10.1002/chem.201102182 22109730

[B111] Kirste A. Schnakenburg G. Waldvogel S. R. (2011b). Anodic coupling of guaiacol derivatives on boron-doped diamond electrodes. Org. Lett. 13, 3126–3129. 10.1021/ol201030g 21608986

[B112] Kissinger P. Heineman W. R. (2018). Laboratory techniques in electroanalytical chemistry, revised and expanded. United States: CRC Press.

[B113] Kong W.-J. Finger L. H. Messinis A. M. Kuniyil R. Oliveira J. C. Ackermann L. (2019). Flow rhodaelectro-catalyzed alkyne annulations by versatile C–H activation: Mechanistic support for rhodium (III/IV). J. Am. Chem. Soc. 141, 17198–17206. 10.1021/jacs.9b07763 31549815

[B114] Kong W. J. Shen Z. Finger L. H. Ackermann L. (2020). Electrochemical access to aza‐polycyclic aromatic hydrocarbons: Rhoda‐electrocatalyzed domino alkyne annulations. Angew. Chem. Int. Ed. 59, 5551–5556. 10.1002/anie.201914775 PMC7155118 31793169

[B115] Kornienko N. Zhao Y. Kley C. S. Zhu C. Kim D. Lin S. (2015). Metal–organic frameworks for electrocatalytic reduction of carbon dioxide. J. Am. Chem. Soc. 137, 14129–14135. 10.1021/jacs.5b08212 26509213

[B116] Kortlever R. Peters I. Balemans C. Kas R. Kwon Y. Mul G. (2016). Palladium–gold catalyst for the electrochemical reduction of CO 2 to C 1–C 5 hydrocarbons. Chem. Commun. 52, 10229–10232. 10.1039/c6cc03717h 27375003

[B117] Kortlever R. Peters I. Koper S. Koper M. T. (2015). Electrochemical CO2 reduction to formic acid at low overpotential and with high faradaic efficiency on carbon-supported bimetallic Pd–Pt nanoparticles. ACS Catal. 5, 3916–3923. 10.1021/acscatal.5b00602

[B118] Kumar G. S. Peshkov A. Brzozowska A. Nikolaienko P. Zhu C. Rueping M. (2020). Nickel‐catalyzed chain‐walking cross‐electrophile coupling of alkyl and aryl halides and olefin hydroarylation enabled by electrochemical reduction. Angew. Chem. Int. Ed. Engl. 59, 6575–6581. 10.1002/ange.201915418 32017340

[B119] Kuttruff C. A. Eastgate M. D. Baran P. S. (2014). Natural product synthesis in the age of scalability. Nat. Prod. Rep. 31, 419–432. 10.1039/c3np70090a 24337165

[B120] Kwon Y. Lum Y. Clark E. L. Ager J. W. Bell A. T. (2016). CO2 electroreduction with enhanced ethylene and ethanol selectivity by nanostructuring polycrystalline copper. ChemElectroChem 3, 1012–1019. 10.1002/celc.201600068

[B121] Lacy D. C. Mccrory C. C. Peters J. C. (2014). Studies of cobalt-mediated electrocatalytic CO2 reduction using a redox-active ligand. Inorg. Chem. 53, 4980–4988. 10.1021/ic403122j 24773584 PMC4033636

[B122] Lai X. L. Shu X. M. Song J. Xu H. C. (2020). Electrophotocatalytic decarboxylative C− H functionalization of heteroarenes. Angew. Chem. Int. Ed. 59, 10626. 10.1002/anie.202002900 32227555

[B123] Lakshmanan S. Murugesan T. (2014). The chlor-alkali process: Work in progress. Clean. Technol. Environ. Policy 16, 225–234. 10.1007/s10098-013-0630-6

[B124] Lan Y. Gai C. Kenis P. J. Lu J. (2014). Electrochemical reduction of carbon dioxide on Cu/CuO core/shell catalysts. ChemElectroChem 1, 1577–1582. 10.1002/celc.201402182

[B125] LarrazáBal G. N. O. Martín A. J. Mitchell S. Hauert R. PéRez-Ramírez J. (2016). Enhanced reduction of CO2 to CO over Cu–in electrocatalysts: Catalyst evolution is the key. ACS Catal. 6, 6265–6274. 10.1021/acscatal.6b02067

[B126] Lee S. Kim D. Lee J. (2015). Electrocatalytic production of C3‐C4 compounds by conversion of CO2 on a chloride‐induced bi‐phasic Cu2O‐Cu catalyst. Angew. Chem. Int. Ed. Engl. 127, 14914–14918. 10.1002/ange.201505730 26473324

[B127] Li C.-J. (2009). Cross-dehydrogenative coupling (CDC): Exploring C− C bond formations beyond functional group transformations. Acc. Chem. Res. 42, 335–344. 10.1021/ar800164n 19220064

[B128] Li J. Liu X. Deng J. Huang Y. Pan Z. Yu Y. (2020). Electrochemical diselenylation of indolizines via intermolecular C–Se formation with 2-methylpyridines, α-bromoketones and diselenides. Chem. Commun. 56, 735–738. 10.1039/c9cc08784b 31840710

[B129] Li L.-J. Jiang Y.-Y. Lam C. M. Zeng C.-C. Hu L.-M. Little R. D. (2015). Aromatic C–H bond functionalization induced by electrochemically *in situ* generated tris (p-bromophenyl) aminium radical cation: Cationic chain reactions of electron-rich aromatics with enamides. J. Org. Chem. 80, 11021–11030. 10.1021/acs.joc.5b02222 26444498

[B130] Li Y.-Q. Yang Q.-L. Fang P. Mei T.-S. Zhang D. (2017). Palladium-catalyzed C (sp2)–H acetoxylation via electrochemical oxidation. Org. Lett. 19, 2905–2908. 10.1021/acs.orglett.7b01138 28537399

[B131] Lim H.-K. Shin H. Goddard W. A. Iii Hwang Y. J. Min B. K. Kim H. (2014). Embedding covalency into metal catalysts for efficient electrochemical conversion of CO2. J. Am. Chem. Soc. 136, 11355–11361. 10.1021/ja503782w 25061918

[B132] Liu K. Wu J. Deng Y. Song C. Song W. Lei A. (2019). Electrochemical C− H/N− H oxidative cross coupling of imidazopyridines with diarylamines to synthesize triarylamine derivatives. ChemElectroChem 6, 4173–4176. 10.1002/celc.201900138

[B133] Liu X. Zhu L. Wang H. He G. Bian Z. (2016). Catalysis performance comparison for electrochemical reduction of CO 2 on Pd–Cu/graphene catalyst. RSC Adv. 6, 38380–38387. 10.1039/c6ra03160a

[B134] Liu Y. Chen S. Quan X. Yu H. (2015). Efficient electrochemical reduction of carbon dioxide to acetate on nitrogen-doped nanodiamond. J. Am. Chem. Soc. 137, 11631–11636. 10.1021/jacs.5b02975 26322741

[B135] Lodh J. Mallick A. Roy S. (2018). Light-driven carbon dioxide reduction coupled with conversion of acetylenic group to ketone by a functional Janus catalyst based on keplerate {Mo_132_}. J. Mat. Chem. A Mat. 6, 20844–20851. 10.1039/c8ta06243a

[B136] Loiudice A. Lobaccaro P. Kamali E. A. Thao T. Huang B. H. Ager J. W. (2016). Tailoring copper nanocrystals towards C2 products in electrochemical CO2 reduction. Angew. Chem. Int. Ed. 55, 5789–5792. 10.1002/anie.201601582 27059162

[B137] Lu Q. Rosen J. Zhou Y. Hutchings G. S. Kimmel Y. C. Chen J. G. (2014a). A selective and efficient electrocatalyst for carbon dioxide reduction. Nat. Commun. 5, 3242–3246. 10.1038/ncomms4242 24476921

[B138] Lu X. Leung D. Y. Wang H. Leung M. K. Xuan J. (2014b). Electrochemical reduction of carbon dioxide to formic acid. ChemElectroChem 1, 836–849. 10.1002/celc.201300206

[B139] Lu X. Tan T. H. Ng Y. H. Amal R. (2016). Highly selective and stable reduction of CO2 to CO by a graphitic carbon nitride/carbon nanotube composite electrocatalyst. Chem. Eur. J. 22, 11991–11996. 10.1002/chem.201601674 27312815

[B140] Lucio A. J. Shaw S. K. (2015). Pyridine and pyridinium electrochemistry on polycrystalline gold electrodes and implications for CO2 reduction. J. Phys. Chem. C 119, 12523–12530. 10.1021/acs.jpcc.5b03355

[B141] Lund H. Hammerich O. (2001). Organic electrochemistry: Revised and expanded. New York: Marcel Dekker.

[B142] Ma C. Fang P. Liu D. Jiao K.-J. Gao P.-S. Qiu H. (2021). Transition metal-catalyzed organic reactions in undivided electrochemical cells. Chem. Sci. 12, 12866–12873. 10.1039/d1sc04011a 34745519 PMC8514006

[B143] Ma C. Fang P. Mei T.-S. (2018). Recent advances in C–H functionalization using electrochemical transition metal catalysis. ACS Catal. 8, 7179–7189. 10.1021/acscatal.8b01697

[B144] Ma M. Djanashvili K. Smith W. A. (2016a). Controllable hydrocarbon formation from the electrochemical reduction of CO2 over Cu nanowire arrays. Angew. Chem. Int. Ed. Engl. 55, 6792–6796. 10.1002/ange.201601282 27098996

[B145] Ma M. Trześniewski B. J. Xie J. Smith W. A. (2016b). Selective and efficient reduction of carbon dioxide to carbon monoxide on oxide‐derived nanostructured silver electrocatalysts. Angew. Chem. Int. Ed. Engl. 128, 9900–9904. 10.1002/ange.201604654 27377237

[B146] Ma S. Lan Y. Perez G. M. Moniri S. Kenis P. J. (2014). Silver supported on titania as an active catalyst for electrochemical carbon dioxide reduction. ChemSusChem 7, 866–874. 10.1002/cssc.201300934 24474718

[B147] Machan C. W. Chabolla S. A. Yin J. Gilson M. K. Tezcan F. A. Kubiak C. P. (2014a). Supramolecular assembly promotes the electrocatalytic reduction of carbon dioxide by Re (I) bipyridine catalysts at a lower overpotential. J. Am. Chem. Soc. 136, 14598–14607. 10.1021/ja5085282 25226161

[B148] Machan C. W. Sampson M. D. Chabolla S. A. Dang T. Kubiak C. P. (2014b). Developing a mechanistic understanding of molecular electrocatalysts for CO2 reduction using infrared spectroelectrochemistry. Organometallics 33, 4550–4559. 10.1021/om500044a

[B149] Mallick A. Roy S. (2018). Visible light driven catalytic gold decorated soft-oxometalate (SOM) based nanomotors for organic pollutant remediation. Nanoscale 10, 12713–12722. 10.1039/c8nr03534b 29946590

[B150] Mann C. K. Grunwald E. (1959). Electroanalytical chemistry. J. Am. Chem. Soc. 81, 5266. 10.1021/ja01528a068

[B151] Manthiram K. Beberwyck B. J. Alivisatos A. P. (2014). Enhanced electrochemical methanation of carbon dioxide with a dispersible nanoscale copper catalyst. J. Am. Chem. Soc. 136, 13319–13325. 10.1021/ja5065284 25137433

[B152] Marcus R. A. (1964). Chemical and electrochemical electron-transfer theory. Annu. Rev. Phys. Chem. 15, 155–196. 10.1146/annurev.pc.15.100164.001103

[B153] Marcus R. A. (1959). On the theory of electrochemical and chemical electron transfer processes. Can. J. Chem. 37, 155–163. 10.1139/v59-022

[B154] Marko J. A. Durgham A. Bretz S. L. Liu W. (2019). Electrochemical benzylic oxidation of C–H bonds. Chem. Commun. 55, 937–940. 10.1039/c8cc08768g 30601486

[B155] Martins G. M. Shirinfar B. Hardwick T. Murtaza A. Ahmed N. (2019). Organic electrosynthesis: Electrochemical alkyne functionalization. Catal. Sci. Technol. 9, 5868–5881. 10.1039/c9cy01312a

[B156] Maurin A. Robert M. (2016). Noncovalent immobilization of a molecular iron-based electrocatalyst on carbon electrodes for selective, efficient CO2-to-CO conversion in water. J. Am. Chem. Soc. 138, 2492–2495. 10.1021/jacs.5b12652 26886174

[B157] Mccann S. D. Stahl S. S. (2015). Copper-catalyzed aerobic oxidations of organic molecules: Pathways for two-electron oxidation with a four-electron oxidant and a one-electron redox-active catalyst. Acc. Chem. Res. 48, 1756–1766. 10.1021/acs.accounts.5b00060 26020118

[B158] Mcnicholas B. J. Blakemore J. D. Chang A. B. Bates C. M. Kramer W. W. Grubbs R. H. (2016). Electrocatalysis of CO2 reduction in brush polymer ion gels. J. Am. Chem. Soc. 138, 11160–11163. 10.1021/jacs.6b08795 27560703

[B159] Meng X. Zhang Y. Luo J. Wang F. Cao X. Huang S. (2020). Electrochemical oxidative oxydihalogenation of alkynes for the synthesis of α, α-dihaloketones. Org. Lett. 22, 1169–1174. 10.1021/acs.orglett.0c00052 31933370

[B160] Mistry H. Varela A. S. Bonifacio C. S. Zegkinoglou I. Sinev I. Choi Y.-W. (2016). Highly selective plasma-activated copper catalysts for carbon dioxide reduction to ethylene. Nat. Commun. 7, 12123–12129. 10.1038/ncomms12123 27356485 PMC4931497

[B161] Moeller K. D. (2016). Anodic olefin coupling reactions: A mechanism driven approach to the development of new synthetic tools. Interface Mag. 25, 53–59. 10.1149/2.f07162if

[B162] Moeller K. D. (1997). “Intramolecular carbon-carbon bond forming reactions at the anode,” in Electrochemistry VI electroorganic synthesis: Bond formation at anode and cathode (Germany: Springer), 49–86.

[B163] Moeller K. D. (2000). Synthetic applications of anodic electrochemistry. Tetrahedron 49, 9527–9554. 10.1016/s0040-4020(00)00840-1

[B164] Möhle S. Zirbes M. Rodrigo E. Gieshoff T. Wiebe A. Waldvogel S. R. (2018). Modern electrochemical aspects for the synthesis of value‐added organic products. Angew. Chem. Int. Ed. 57, 6018–6041. 10.1002/anie.201712732 PMC6001547 29359378

[B165] MorlanéS N. Takanabe K. Rodionov V. (2016). Simultaneous reduction of CO2 and splitting of H2O by a single immobilized cobalt phthalocyanine electrocatalyst. ACS Catal. 6, 3092–3095. 10.1021/acscatal.6b00543

[B166] Morofuji T. Shimizu A. Yoshida J.-I. (2014). Direct C–N coupling of imidazoles with aromatic and benzylic compounds via electrooxidative C–H functionalization. J. Am. Chem. Soc. 136, 4496–4499. 10.1021/ja501093m 24625055

[B167] Morofuji T. Shimizu A. Yoshida J.-I. (2015). Electrochemical intramolecular C-H amination: Synthesis of benzoxazoles and benzothiazoles. Chem. Eur. J. 21, 3211–3214. 10.1002/chem.201406398 25641711

[B168] Morofuji T. Shimizu A. Yoshida J. I. (2012). Metal-and chemical-oxidant-free C H/C H cross-coupling of aromatic compounds: The use of radical-cation pools. Angew. Chem. Int. Ed. Engl. 124, 7371–7374. 10.1002/ange.201202788 22689181

[B169] Nakata K. Ozaki T. Terashima C. Fujishima A. Einaga Y. (2014). High-yield electrochemical production of formaldehyde from CO2 and seawater. Angew. Chem. Int. Ed. 53, 871–874. 10.1002/anie.201308657 24281847

[B170] Narayan R. Matcha K. Antonchick A. P. (2015). Metal-free oxidative C-C bond formation through C-H bond functionalization H bond functionalization. Chem. Eur. J. 21, 14678–14693. 10.1002/chem.201502005- 26239615

[B171] Nikolaienko P. Jentsch M. Kale A. P. Cai Y. Rueping M. (2019). Electrochemical and scalable dehydrogenative C (sp 3)− H amination via remote hydrogen atom transfer in batch and continuous flow. Chem. Eur. J. 25, 7177–7184. 10.1002/chem.201806092 30861204

[B172] Nokami T. Ohata K. Inoue M. Tsuyama H. Shibuya A. Soga K. (2008). Iterative molecular assembly based on the cation-pool method. Convergent synthesis of dendritic molecules. J. Am. Chem. Soc. 130, 10864–10865. 10.1021/ja803487q 18661982

[B173] Nutting J. E. Rafiee M. Stahl S. S. (2018). Tetramethylpiperidine N-oxyl (TEMPO), phthalimide N-oxyl (PINO), and related N-oxyl species: Electrochemical properties and their use in electrocatalytic reactions. Chem. Rev. 118, 4834–4885. 10.1021/acs.chemrev.7b00763 29707945 PMC6284524

[B174] O'brien A. G. Maruyama A. Inokuma Y. Fujita M. Baran P. S. Blackmond D. G. (2014). Radical C-H functionalization of heteroarenes under electrochemical control H functionalization of heteroarenes under electrochemical control. Angew. Chem. Int. Ed. 53, 11868–11871. 10.1002/anie.201407948- PMC4214156 25209429

[B175] Ogawa K. A. Boydston A. J. (2015). Recent developments in organocatalyzed electroorganic chemistry. Chem. Lett. 44, 10–16. 10.1246/cl.140915

[B176] Ogibin Y. N. Elinson M. N. Nikishin G. I. (2009). Mediator oxidation systems in organic electrosynthesis. Russ. Chem. Rev. 78, 89–140. 10.1070/rc2009v078n02abeh003886

[B177] Oh S. Gallagher J. R. Miller J. T. Surendranath Y. (2016). Graphite-conjugated rhenium catalysts for carbon dioxide reduction. J. Am. Chem. Soc. 138, 1820–1823. 10.1021/jacs.5b13080 26804469

[B178] Oh Y. Vrubel H. Guidoux S. Hu X. (2014). Electrochemical reduction of CO 2 in organic solvents catalyzed by MoO 2. Chem. Commun. 50, 3878–3881. 10.1039/c3cc49262a 24589502

[B179] Okajima M. Suga S. Itami K. Yoshida J.-I. (2005). Cation pool” method based on C− C bond dissociation. Effective generation of monocations and dications. J. Am. Chem. Soc. 127, 6930–6931. 10.1021/ja050414y 15884918

[B180] Osadchuk I. Tamm T. Ahlquist M. R. S. (2016). Reduced state of iridium PCP pincer complexes in electrochemical CO2 hydrogenation. ACS Catal. 6, 3834–3839. 10.1021/acscatal.6b01233

[B181] Poh H. L. Sofer Z. Luxa J. Pumera M. (2014). Transition metal‐depleted graphenes for electrochemical applications via reduction of CO2 by lithium. Small 10, 1529–1535. 10.1002/smll.201303002 24344051

[B183] Pollok D. Waldvogel S. R. (2020). Electro-organic synthesis–a 21 st century technique. Chem. Sci. 11, 12386–12400. 10.1039/d0sc01848a 34123227 PMC8162804

[B184] Popp F. D. Schultz H. P. (1962). Electrolytic reduction of organic compounds. Chem. Rev. 62, 19–40. 10.1021/cr60215a002

[B185] Raciti D. Livi K. J. Wang C. (2015). Highly dense Cu nanowires for low-overpotential CO2 reduction. Nano Lett. 15, 6829–6835. 10.1021/acs.nanolett.5b03298 26352048

[B186] Rafiee M. Konz Z. M. Graaf M. D. Koolman H. F. Stahl S. S. (2018). Electrochemical oxidation of alcohols and aldehydes to carboxylic acids catalyzed by 4-acetamido-TEMPO: An alternative to “Anelli” and “Pinnick” oxidations. ACS Catal. 8, 6738–6744. 10.1021/acscatal.8b01640

[B187] Rafiee M. Miles K. C. Stahl S. S. (2015). Electrocatalytic alcohol oxidation with TEMPO and bicyclic nitroxyl derivatives: Driving force trumps steric effects. J. Am. Chem. Soc. 137, 14751–14757. 10.1021/jacs.5b09672 26505317 PMC4863653

[B188] Rao H. Schmidt L. C. Bonin J. Robert M. (2017). Visible-light-driven methane formation from CO 2 with a molecular iron catalyst. Nature 548, 74–77. 10.1038/nature23016 28723895

[B189] Rasul S. Anjum D. H. Jedidi A. Minenkov Y. Cavallo L. Takanabe K. (2015). A highly selective copper–indium bimetallic electrocatalyst for the electrochemical reduction of aqueous CO2 to CO. Angew. Chem. Int. Ed. Engl. 127, 2174–2178. 10.1002/ange.201410233 25537315

[B190] Ren D. Deng Y. Handoko A. D. Chen C. S. Malkhandi S. Yeo B. S. (2015). Selective electrochemical reduction of carbon dioxide to ethylene and ethanol on copper (I) oxide catalysts. ACS Catal. 5, 2814–2821. 10.1021/cs502128q

[B191] Reuillard B. Ly K. H. Rosser T. E. Kuehnel M. F. Zebger I. Reisner E. (2017). Tuning product selectivity for aqueous CO2 reduction with a Mn (bipyridine)-pyrene catalyst immobilized on a carbon nanotube electrode. J. Am. Chem. Soc. 139, 14425–14435. 10.1021/jacs.7b06269 28885841 PMC5649446

[B192] Riplinger C. Carter E. A. (2015). Influence of weak Brønsted acids on electrocatalytic CO2 reduction by manganese and rhenium bipyridine catalysts. ACS Catal. 5, 900–908. 10.1021/cs501687n

[B193] Roberts F. S. Kuhl K. P. Nilsson A. (2015). High selectivity for ethylene from carbon dioxide reduction over copper nanocube electrocatalysts. Angew. Chem. Int. Ed. Engl. 127, 5179–5182. 10.1002/anie.201412214 25728325

[B194] Rosen B. R. Werner E. W. O’brien A. G. Baran P. S. (2014). Total synthesis of dixiamycin B by electrochemical oxidation. J. Am. Chem. Soc. 136, 5571–5574. 10.1021/ja5013323 24697810 PMC4004216

[B195] Rosen J. Hutchings G. S. Lu Q. Forest R. V. Moore A. Jiao F. (2015a). Electrodeposited Zn dendrites with enhanced CO selectivity for electrocatalytic CO2 reduction. ACS Catal. 5, 4586–4591. 10.1021/acscatal.5b00922

[B196] Rosen J. Hutchings G. S. Lu Q. Rivera S. Zhou Y. Vlachos D. G. (2015b). Mechanistic insights into the electrochemical reduction of CO2 to CO on nanostructured Ag surfaces. ACS Catal. 5, 4293–4299. 10.1021/acscatal.5b00840

[B197] Roy N. Shibano Y. Terashima C. Katsumata K. I. Nakata K. Kondo T. (2016). Ionic liquid assisted selective and controlled electrochemical CO2 reduction at cu-modified boron-doped diamond electrode. ChemElectroChem 3, 1044–1047. 10.1002/celc.201600105

[B198] Ruan Z. Huang Z. Xu Z. Mo G. Tian X. Yu X.-Y. (2019). Catalyst-free, direct electrochemical tri-and difluoroalkylation/cyclization: Access to functionalized oxindoles and quinolinones. Org. Lett. 21, 1237–1240. 10.1021/acs.orglett.9b00361 30730146

[B199] Ryan M. C. Whitmire L. D. Mccann S. D. Stahl S. S. (2019). Copper/TEMPO redox redux: Analysis of PCET oxidation of TEMPOH by copper (II) and the reaction of TEMPO with copper (I). Inorg. Chem. 58, 10194–10200. 10.1021/acs.inorgchem.9b01326 31283193 PMC7641458

[B200] Ryland B. L. Mccann S. D. Brunold T. C. Stahl S. S. (2014). Mechanism of alcohol oxidation mediated by copper (II) and nitroxyl radicals. J. Am. Chem. Soc. 136, 12166–12173. 10.1021/ja5070137 25090238 PMC4354946

[B201] Ryland B. L. Stahl S. S. (2014). Practical aerobic oxidations of alcohols and amines with homogeneous copper/TEMPO and related catalyst systems. Angew. Chem. Int. Ed. 53, 8824–8838. 10.1002/anie.201403110 PMC4165639 25044821

[B202] Sarfraz S. Garcia-Esparza A. T. Jedidi A. Cavallo L. Takanabe K. (2016). Cu–Sn bimetallic catalyst for selective aqueous electroreduction of CO2 to CO. ACS Catal. 6, 2842–2851. 10.1021/acscatal.6b00269

[B203] Sato S. Saita K. Sekizawa K. Maeda S. Morikawa T. (2018). Low-energy electrocatalytic CO2 reduction in water over Mn-complex catalyst electrode aided by a nanocarbon support and K+ cations. ACS Catal. 8, 4452–4458. 10.1021/acscatal.8b01068

[B204] Sauermann N. Mei R. Ackermann L. (2018). Electrochemical C− H amination by cobalt catalysis in a renewable solvent. Angew. Chem. Int. Ed. Engl. 57, 5184–5188. 10.1002/ange.201802206 29509336

[B205] Sauermann N. Meyer T. H. Tian C. Ackermann L. (2017). Electrochemical cobalt-catalyzed C–H oxygenation at room temperature. J. Am. Chem. Soc. 139, 18452–18455. 10.1021/jacs.7b11025 29149561

[B206] Sawyer D. T. Sobkowiak A. Roberts J. L. (1995). Electrochemistry for chemists. United States: Wiley.

[B207] Schäfer H. J. (1981). Anodic and cathodic CC‐bond formation. Angew. Chem. Int. Ed. Engl. 20, 911–934. 10.1002/anie.198109111

[B208] Schäfer H. J. (2014). Carbon–carbon bond formation via electron transfer: Anodic coupling. ChemCatChem 6, 2792–2795. 10.1002/cctc.201402366

[B209] Schäfer H. J. (2011). Contributions of organic electrosynthesis to green chemistry. Comptes Rendus Chim. 14, 745–765. 10.1016/j.crci.2011.01.002

[B210] Schäfer H. J. Harenbrock M. Klocke E. Plate M. Weiper-Idelmann A. (2007). Electrolysis for the benign conversion of renewable feedstocks. Pure Appl. Chem. 79, 2047–2057. 10.1351/pac200779112047

[B211] Schulz L. Enders M. Elsler B. Schollmeyer D. Dyballa K. M. Franke R. (2017). Reagent‐and metal‐free anodic C− C cross‐coupling of aniline derivatives. Angew. Chem. Int. Ed. 56, 4877–4881. 10.1002/anie.201612613 28252240

[B212] Schulz L. Waldvogel S. R. (2019). Solvent control in electro-organic synthesis. Synlett 30, 275–286. 10.1055/s-0037-1610303

[B213] Sen S. Liu D. Palmore G. T. R. (2014). Electrochemical reduction of CO2 at copper nanofoams. ACS Catal. 4, 3091–3095. 10.1021/cs500522g

[B214] Senboku H. Hayama M. Matsuno H. (2022). Electrochemical Friedel–Crafts-type amidomethylation of arenes by a novel electrochemical oxidation system using a quasi-divided cell and trialkylammonium tetrafluoroborate. Beilstein J. Org. Chem. 18, 1040–1046. 10.3762/bjoc.18.105 36105724 PMC9443422

[B215] Sequeira C. Santos D. (2009). Electrochemical routes for industrial synthesis. J. Braz. Chem. Soc. 20, 387–406. 10.1590/s0103-50532009000300002

[B216] Shao X. Zheng Y. Tian L. Martín-Torres I. Echavarren A. M. Wang Y. (2019). Decarboxylative c_sp<sup>3</sup>_–N bond formation by electrochemical oxidation of amino acids. Org. Lett. 21, 9262–9267. 10.1021/acs.orglett.9b03696 31661284

[B217] Sharma P. P. Wu J. Yadav R. M. Liu M. Wright C. J. Tiwary C. S. (2015). Nitrogen‐doped carbon nanotube arrays for high‐efficiency electrochemical reduction of CO2: On the understanding of defects, defect density, and selectivity. Angew. Chem. Int. Ed. Engl. 127, 13905–13909. 10.1002/ange.201506062 26404732

[B218] Shen J. Kolb M. J. Gottle A. J. Koper M. T. (2016). DFT study on the mechanism of the electrochemical reduction of CO2 catalyzed by cobalt porphyrins. J. Phys. Chem. C 120, 15714–15721. 10.1021/acs.jpcc.5b10763

[B219] Shen J. Kortlever R. Kas R. Birdja Y. Y. Diaz-Morales O. Kwon Y. (2015). Electrocatalytic reduction of carbon dioxide to carbon monoxide and methane at an immobilized cobalt protoporphyrin. Nat. Commun. 6, 8177–8178. 10.1038/ncomms9177 26324108 PMC4569799

[B220] Shi C. Chan K. Yoo J. S. Nørskov J. K. (2016). Barriers of electrochemical CO2 reduction on transition metals. Org. Process Res. Dev. 20, 1424–1430. 10.1021/acs.oprd.6b00103

[B221] Shono T. (1984). Electroorganic chemistry in organic synthesis. Tetrahedron 40, 811–850. 10.1016/s0040-4020(01)91472-3

[B222] Singh M. R. Kwon Y. Lum Y. Ager J. W. Iii Bell A. T. (2016). Hydrolysis of electrolyte cations enhances the electrochemical reduction of CO2 over Ag and Cu. J. Am. Chem. Soc. 138, 13006–13012. 10.1021/jacs.6b07612 27626299

[B223] Song Y. Peng R. Hensley D. K. Bonnesen P. V. Liang L. Wu Z. (2016). High‐selectivity electrochemical conversion of CO2 to ethanol using a copper nanoparticle/N‐doped graphene electrode. ChemistrySelect 1, 6055–6061. 10.1002/slct.201601169

[B224] Sperry J. B. Wright D. L. (2006). The application of cathodic reductions and anodic oxidations in the synthesis of complex molecules. Chem. Soc. Rev. 35, 605–621. 10.1039/b512308a 16791332

[B225] Stanton C. J. Iii Vandezande J. E. Majetich G. F. Schaefer H. F. Iii Agarwal J. (2016). Mn-NHC electrocatalysts: Increasing π acidity lowers the reduction potential and increases the turnover frequency for CO2 reduction. Inorg. Chem. 55, 9509–9512. 10.1021/acs.inorgchem.6b01657 27636737

[B226] Steckhan E. (1986). Indirect electroorganic syntheses—a modern chapter of organic electrochemistry [new synthetic methods (59)]. Angew. Chem. Int. Ed. Engl. 25, 683–701. 10.1002/anie.198606831

[B227] Steckhan E. (1987). “Organic syntheses with electrochemically regenerable redox systems,” in Electrochemistry I (Germany: Springer), 1–69.

[B228] Steves J. E. Stahl S. S. (2013). Copper (I)/ABNO-catalyzed aerobic alcohol oxidation: Alleviating steric and electronic constraints of Cu/TEMPO catalyst systems. J. Am. Chem. Soc. 135, 15742–15745. 10.1021/ja409241h 24128057 PMC6346749

[B229] Studt F. Sharafutdinov I. Abild-Pedersen F. Elkjær C. F. Hummelshøj J. S. Dahl S. (2014). Discovery of a Ni-Ga catalyst for carbon dioxide reduction to methanol. Nat. Chem. 6, 320–324. 10.1038/nchem.1873 24651199

[B230] Su P. Iwase K. Nakanishi S. Hashimoto K. Kamiya K. (2016). Nickel‐nitrogen‐modified graphene: An efficient electrocatalyst for the reduction of carbon dioxide to carbon monoxide. Small 12, 6083–6089. 10.1002/smll.201602158 27634486

[B231] Sun L. Ramesha G. K. Kamat P. V. Brennecke J. F. (2014). Switching the reaction course of electrochemical CO2 reduction with ionic liquids. Langmuir 30, 6302–6308. 10.1021/la5009076 24851903

[B232] Sun X. Ma H.-X. Mei T.-S. Fang P. Hu Y. (2019). Electrochemical radical formyloxylation–bromination, − chlorination, and− trifluoromethylation of alkenes. Org. Lett. 21, 3167–3171. 10.1021/acs.orglett.9b00867 30995058

[B233] Sun X. Zhu Q. Kang X. Liu H. Qian Q. Zhang Z. (2016). Molybdenum–bismuth bimetallic chalcogenide nanosheets for highly efficient electrocatalytic reduction of carbon dioxide to methanol. Angew. Chem. Int. Ed. Engl. 128, 6883–6887. 10.1002/ange.201603034 27098284

[B234] Sun Y. Li X. Yang M. Xu W. Xie J. Ding M. (2020). Highly selective electrocatalytic oxidation of benzyl C–H using water as safe and sustainable oxygen source. Green Chem. 22, 7543–7551. 10.1039/d0gc01871f

[B235] Tang S. Wang D. Liu Y. Zeng L. Lei A. (2018a). Cobalt-catalyzed electrooxidative CH/NH [4+ 2] annulation with ethylene or ethyne. Nat. Commun. 9, 798–804. 10.1038/s41467-018-03246-4 29476057 PMC5824839

[B236] Tang S. Wang S. Liu Y. Cong H. Lei A. (2018b). Electrochemical oxidative C− H amination of phenols: Access to triarylamine derivatives. Angew. Chem. Int. Ed. Engl. 130, 4827–4831. 10.1002/ange.201800240 29498166

[B237] Therrien J. A. Wolf M. O. Patrick B. O. (2014). Electrocatalytic reduction of CO2 with palladium bis-N-heterocyclic carbene pincer complexes. Inorg. Chem. 53, 12962–12972. 10.1021/ic502056w 25337973

[B238] Therrien J. A. Wolf M. O. Patrick B. O. (2015). Polyannulated bis (N-heterocyclic carbene) palladium pincer complexes for electrocatalytic CO2 reduction. Inorg. Chem. 54, 11721–11732. 10.1021/acs.inorgchem.5b01698 26624491

[B239] Tian C. Massignan L. Meyer T. H. Ackermann L. (2018). Electrochemical C− H/N− H activation by water‐tolerant cobalt catalysis at room temperature. Angew. Chem. Int. Ed. Engl. 130, 2407–2411. 10.1002/ange.201712647 29316187

[B240] Tojo G. Fernández M. (2010a). Basic reactions in organic synthesis. *Oxidation of primary Alcohols to carboxylic acids* . New York, NY: Springer.

[B241] Tojo G. Fernández M. (2010b). Oxidation of primary alcohols to carboxylic acids basic reactions in organic synthesis. New York: Springer.

[B242] Torelli D. A. Francis S. A. Crompton J. C. Javier A. Thompson J. R. Brunschwig B. S. (2016). Nickel–gallium-catalyzed electrochemical reduction of CO2 to highly reduced products at low overpotentials. ACS Catal. 6, 2100–2104. 10.1021/acscatal.5b02888

[B243] Tory J. Setterfield-Price B. Dryfe R. A. Hartl F. (2015). [M(CO)_4_(2, 2′-bipyridine)] (M=Cr, Mo, W) complexes as efficient catalysts for electrochemical reduction of CO_2_ at a gold electrode. ChemElectroChem 2, 213–217. 10.1002/celc.201402282

[B244] Varela A. S. Ranjbar Sahraie N. Steinberg J. Ju W. Oh H. S. Strasser P. (2015). Metal‐doped nitrogenated carbon as an efficient catalyst for direct CO2 electroreduction to CO and hydrocarbons. Angew. Chem. Int. Ed. Engl. 127, 10758–10762. 10.1002/anie.201502099 26227677

[B245] Vijh A. Conway B. (1967). Electrode kinetic aspects of the Kolbe reaction. Chem. Rev. 67, 623–664. 10.1021/cr60250a003

[B246] Vock S. Tschulik K. Uhlemann M. Hengst C. Fähler S. Schultz L. (2015). Magnetostatic nearest neighbor interactions in a Co48Fe52 nanowire array probed by in-field magnetic force microscopy. J. Appl. Phys. 118, 233901. 10.1063/1.4937275

[B247] Vollmer M. V. Machan C. W. Clark M. L. Antholine W. E. Agarwal J. Schaefer H. F. Iii (2015). Synthesis, spectroscopy, and electrochemistry of (α-Diimine) M (CO) 3Br, M= Mn, Re, complexes: Ligands isoelectronic to bipyridyl show differences in CO2 reduction. Organometallics 34, 3–12. 10.1021/om500838z PMC4399245 25892841

[B248] Waldvogel S. R. Dörr M. Röckl J. Rein J. Schollmeyer D. (2020). Electrochemical C‐H functionalization of (hetero) arenes–optimized by DoE. Chem. Eur. J. 26, 10195–10198. 10.1002/chem.202001171 32232873 PMC7496267

[B249] Waldvogel S. R. Lips S. Selt M. Riehl B. Kampf C. J. (2018). Electrochemical arylation reaction. Chem. Rev. 118, 6706–6765. 10.1021/acs.chemrev.8b00233 29963856

[B250] Waldvogel S. R. Selt M. (2016). Electrochemical allylic oxidation of olefins: Sustainable and safe. Angew. Chem. Int. Ed. 55, 12578–12580. 10.1002/anie.201606727 27528371

[B251] Walsh J. J. Neri G. Smith C. L. Cowan A. J. (2014). Electrocatalytic CO 2 reduction with a membrane supported manganese catalyst in aqueous solution. Chem. Commun. 50, 12698–12701. 10.1039/c4cc06404f 25204759

[B252] Walsh J. J. Smith C. L. Neri G. Whitehead G. F. Robertson C. M. Cowan A. J. (2015). Improving the efficiency of electrochemical CO2 reduction using immobilized manganese complexes. Faraday Discuss. 183, 147–160. 10.1039/c5fd00071h 26375151

[B253] Wang F. Rafiee M. Stahl S. S. (2018). Electrochemical functional‐group‐tolerant shono‐type oxidation of cyclic carbamates enabled by aminoxyl mediators. Angew. Chem. Int. Ed. Engl. 130, 6796–6800. 10.1002/ange.201803539 PMC6201259 29659129

[B254] Wang F. Stahl S. S. (2019). Merging photochemistry with electrochemistry: Functional‐group tolerant electrochemical amination of C (sp3)− H bonds. Angew. Chem. Int. Ed. Engl. 58, 6451–6456. 10.1002/ange.201813960 PMC6482061 30763466

[B255] Wang J.-H. Lei T. Nan X.-L. Wu H.-L. Li X.-B. Chen B. (2019a). Regioselective ortho amination of an aromatic C–H bond by trifluoroacetic acid via electrochemistry. Org. Lett. 21, 5581–5585. 10.1021/acs.orglett.9b01910 31276420

[B256] Wang P. Yang Z. Wu T. Xu C. Wang Z. Lei A. (2019b). Electrochemical oxidative C (sp3)− H/N− H cross‐coupling for N‐mannich bases with hydrogen evolution. ChemSusChem 12, 3073–3077. 10.1002/cssc.201802676 30548917

[B257] Wang Y. Deng L. Wang X. Wu Z. Wang Y. Pan Y. (2019c). Electrochemically promoted nickel-catalyzed carbon–sulfur bond formation. ACS Catal. 9, 1630–1634. 10.1021/acscatal.8b04633

[B258] Wang Z. Yang G. Zhang Z. Jin M. Yin Y. (2016). Selectivity on etching: Creation of high-energy facets on copper nanocrystals for CO2 electrochemical reduction. ACS Nano 10, 4559–4564. 10.1021/acsnano.6b00602 26974506

[B259] Wannakao S. Artrith N. Limtrakul J. Kolpak A. M. (2015). Engineering transition-metal-coated tungsten carbides for efficient and selective electrochemical reduction of CO2 to methane. ChemSusChem 8, 2745–2751. 10.1002/cssc.201500245 26219085

[B260] Weetal H. H. Forsyth B. D. Hertl W. (1985). A direct fuel cell for the production of electricity from lignin. Biotechnol. Bioeng. 27, 972–979. 10.1002/bit.260270707 18553766

[B261] Weibel D. B. Boulatov R. Lee A. Ferrigno R. Whitesides G. M. (2005). Modeling the anodic half‐cell of a low‐temperature coal fuel cell. Angew. Chem. Int. Ed. Engl. 117, 5682–5686. 10.1002/anie.200501192 16086348

[B262] Weng Z. Jiang J. Wu Y. Wu Z. Guo X. Materna K. L. (2016). Electrochemical CO2 reduction to hydrocarbons on a heterogeneous molecular Cu catalyst in aqueous solution. J. Am. Chem. Soc. 138, 8076–8079. 10.1021/jacs.6b04746 27310487

[B263] White R. E. (2012). Electrochemical cell design. Berlin, Germany: Springer Science & Business Media.

[B264] Wiebe A. Gieshoff T. Möhle S. Rodrigo E. Zirbes M. Waldvogel S. R. (2018). Electrifying organic synthesis. Angew. Chem. Int. Ed. 57, 5594–5619. 10.1002/anie.201711060 PMC5969240 29292849

[B265] Wiebe A. Lips S. Schollmeyer D. Franke R. Waldvogel S. R. (2017). Single and twofold metal‐and reagent‐free anodic C− C cross‐coupling of phenols with thiophenes. Angew. Chem. Int. Ed. 56, 14727–14731. 10.1002/anie.201708946 28967700

[B266] Won D. H. Choi C. H. Chung J. Chung M. W. Kim E. H. Woo S. I. (2015). Rational design of a hierarchical tin dendrite electrode for efficient electrochemical reduction of CO2. ChemSusChem 8, 3092–3098. 10.1002/cssc.201500694 26219092

[B267] Won D. H. Shin H. Koh J. Chung J. Lee H. S. Kim H. (2016). Highly efficient, selective, and stable CO2 electroreduction on a hexagonal Zn catalyst. Angew. Chem. Int. Ed. Engl. 55, 9443–9446. 10.1002/ange.201602888 27352078

[B268] Wu J. Liu M. Sharma P. P. Yadav R. M. Ma L. Yang Y. (2016a). Incorporation of nitrogen defects for efficient reduction of CO2 via two-electron pathway on three-dimensional graphene foam. Nano Lett. 16, 466–470. 10.1021/acs.nanolett.5b04123 26651056

[B269] Wu J. Risalvato F. G. Ma S. Zhou X.-D. (2014). Electrochemical reduction of carbon dioxide III. The role of oxide layer thickness on the performance of Sn electrode in a full electrochemical cell. J. Mat. Chem. A 2, 1647–1651. 10.1039/c3ta13544f

[B270] Wu J. Yadav R. M. Liu M. Sharma P. P. Tiwary C. S. Ma L. (2015). Achieving highly efficient, selective, and stable CO2 reduction on nitrogen-doped carbon nanotubes. ACS Nano 9, 5364–5371. 10.1021/acsnano.5b01079 25897553

[B271] Wu W. Liu W. Mu W. Deng Y. (2016b). Polyoxymetalate liquid-catalyzed polyol fuel cell and the related photoelectrochemical reaction mechanism study. J. Power Sources 318, 86–92. 10.1016/j.jpowsour.2016.03.074

[B272] Xiang D. Magana D. Dyer R. B. (2014). CO2 reduction catalyzed by mercaptopteridine on glassy carbon. J. Am. Chem. Soc. 136, 14007–14010. 10.1021/ja5081103 25259884

[B273] Xiao H. Cheng T. Goddard W. A. Iii Sundararaman R. (2016). Mechanistic explanation of the pH dependence and onset potentials for hydrocarbon products from electrochemical reduction of CO on Cu (111). J. Am. Chem. Soc. 138, 483–486. 10.1021/jacs.5b11390 26716884

[B274] Xu F. Li Y.-J. Huang C. Xu H.-C. (2018). Ruthenium-catalyzed electrochemical dehydrogenative alkyne annulation. ACS Catal. 8, 3820–3824. 10.1021/acscatal.8b00373

[B275] Yadav V. Purkait M. (2015). Electrochemical studies for CO2 reduction using synthesized Co3O4 (Anode) and Cu2O (Cathode) as electrocatalysts. Energy fuels. 29, 6670–6677. 10.1021/acs.energyfuels.5b01656

[B276] Yang H. Shen Y. Xiao Z. Liu C. Yuan K. Ding Y. (2020). The direct trifluoromethylsilylation and cyanosilylation of aldehydes via an electrochemically induced intramolecular pathway. Chem. Commun. 56, 2435–2438. 10.1039/c9cc08975f 31996885

[B277] Yang Q.-L. Wang X.-Y. Lu J.-Y. Zhang L.-P. Fang P. Mei T.-S. (2018a). Copper-catalyzed electrochemical C–H amination of arenes with secondary amines. J. Am. Chem. Soc. 140, 11487–11494. 10.1021/jacs.8b07380 30165030

[B278] Yang Q.-L. Xing Y.-K. Wang X.-Y. Ma H.-X. Weng X.-J. Yang X. (2019). Electrochemistry-enabled Ir-catalyzed vinylic C–H functionalization. J. Am. Chem. Soc. 141, 18970–18976. 10.1021/jacs.9b11915 31714747

[B279] Yang Q. L. Fang P. Mei T. S. (2018b). Recent advances in organic electrochemical C—H functionalization. Chin. J. Chem. 36, 338–352. 10.1002/cjoc.201700740

[B280] Yoshida J.-I. Kataoka K. Horcajada R. Nagaki A. (2008). Modern strategies in electroorganic synthesis. Chem. Rev. 108, 2265–2299. 10.1021/cr0680843 18564879

[B281] Yuan Y. Chen Y. Tang S. Huang Z. Lei A. (2018). Electrochemical oxidative oxysulfenylation and aminosulfenylation of alkenes with hydrogen evolution. Sci. Adv. 4, eaat5312. 10.1126/sciadv.aat5312 30083610 PMC6070360

[B282] Zhang L. Zhang G. Wang P. Li Y. Lei A. (2018a). Electrochemical oxidation with lewis-acid catalysis leads to trifluoromethylative difunctionalization of alkenes using CF3SO2Na. Org. Lett. 20, 7396–7399. 10.1021/acs.orglett.8b03081 30461286

[B283] Zhang S. Kang P. Meyer T. J. (2014a). Nanostructured tin catalysts for selective electrochemical reduction of carbon dioxide to formate. J. Am. Chem. Soc. 136, 1734–1737. 10.1021/ja4113885 24417470

[B284] Zhang S. Kang P. Ubnoske S. Brennaman M. K. Song N. House R. L. (2014b). Polyethylenimine-enhanced electrocatalytic reduction of CO2 to formate at nitrogen-doped carbon nanomaterials. J. Am. Chem. Soc. 136, 7845–7848. 10.1021/ja5031529 24779427

[B285] Zhang X. Wang C. Jiang H. Sun L. (2018b). Convenient synthesis of selenyl-indoles via iodide ion-catalyzed electrochemical C–H selenation. Chem. Commun. 54, 8781–8784. 10.1039/c8cc04543g 30035282

[B286] Zhang X. Wu Z. Zhang X. Li L. Li Y. Xu H. (2017). Highly selective and active CO 2 reduction electrocatalysts based on cobalt phthalocyanine/carbon nanotube hybrid structures. Nat. Commun. 8, 14675–14678. 10.1038/ncomms14675 28272403 PMC5344970

[B287] Zhang Y.-J. Sethuraman V. Michalsky R. Peterson A. A. (2014c). Competition between CO2 reduction and H2 evolution on transition-metal electrocatalysts. ACS Catal. 4, 3742–3748. 10.1021/cs5012298

[B288] Zhang Z. Chi M. Veith G. M. Zhang P. Lutterman D. A. Rosenthal J. (2016). Rational design of Bi nanoparticles for efficient electrochemical CO2 reduction: The elucidation of size and surface condition effects. ACS Catal. 6, 6255–6264. 10.1021/acscatal.6b01297

[B289] Zhang Z. Zhang L. Cao Y. Li F. Bai G. Liu G. (2019). Mn-mediated electrochemical trifluoromethylation/C (sp2)–H functionalization cascade for the synthesis of azaheterocycles. Org. Lett. 21, 762–766. 10.1021/acs.orglett.8b04010 30672710

[B290] Zheng Q. Liu C. F. Chen J. Rao G. W. (2020). C–H functionalization of aromatic amides. Adv. Synth. Catal. 362, 1406–1446. 10.1002/adsc.201901158

[B291] Zhu W. Zhang Y.-J. Zhang H. Lv H. Li Q. Michalsky R. (2014). Active and selective conversion of CO2 to CO on ultrathin Au nanowires. J. Am. Chem. Soc. 136, 16132–16135. 10.1021/ja5095099 25380393

[B292] Zou Z. Zhang W. Wang Y. Kong L. Karotsis G. Wang Y. (2019). Electrochemically promoted fluoroalkylation–distal functionalization of unactivated alkenes. Org. Lett. 21, 1857–1862. 10.1021/acs.orglett.9b00444 30817165

